# Design of Natterins-based peptides improves antimicrobial and antiviral activities

**DOI:** 10.1016/j.btre.2024.e00867

**Published:** 2024-11-28

**Authors:** Gabrielle L. de Cena, Dayane B. Tada, Danilo B.M. Lucchi, Tiago A.A. Santos, Montserrat Heras, Maria Juliano, Carla Torres Braconi, Miguel A.R.B. Castanho, Mônica Lopes-Ferreira, Katia Conceição

**Affiliations:** a Laboratory of Peptide Biochemistry, Universidade Federal de São Paulo (UNIFESP), São José dos Campos, Brazil; b Laboratory of Nanomaterials and Nanotoxicology, Universidade Federal de São Paulo (UNIFESP), São José dos Campos, Brazil; c Department of Microbiology, Immunology and Parasitology, Escola Paulista de Medicina (UNIFESP), São Paulo, Brazil; d Instituto de Medicina Molecular, Faculdade de Medicina da Universidade de Lisboa, Av. Professor Egas Moniz, 1649-028 Lisboa, Portugal; e Departament de Química, Universitat de Girona, Campus Montilivi, 17071 Girona, Spain; f Department of Biophysics, Escola Paulista de Medicina (UNIFESP), São Paulo, Brazil; g Immunoregulation Unit, Laboratory of Applied Toxinology (CeTICs/FAPESP), Butantan Institute, São Paulo 05503900, Brazil

**Keywords:** Bioactive peptides, Antimicrobial peptides, Cell penetrating peptides, *In silico* prediction, ADMET

## Abstract

The biochemical analysis of animal venoms has been intensifying over the years, enabling the prediction of new molecules derived from toxins, harnessing the therapeutic potential of these molecules. From the venom of the fish *Thalassophryne nattereri*, using *in silico* methods for predicting antimicrobial and cell-penetrating peptides, two peptides from Natterins with promising characteristics were synthesized and subjected to *in vitro* and *in vivo* analysis. The peptides were subjected to stability tests and antimicrobial assays, cytotoxicity in murine fibroblast cells, antiviral assays against the Chikungunya virus, and the toxicity on *G. mellonella* was also evaluated. The findings underscore the peptides' robust stability under varying temperatures and pH conditions and resistance to proteolytic degradation. The peptides demonstrated significant antimicrobial efficacy, minimal cytotoxicity, and low hemolytic activity. Although their antiviral efficacy was limited, they showed potential at specific stages of viral replication. The *in vivo* toxicity tests indicated a favorable safety profile. These findings suggest that this approach can aid in the development of antimicrobial agents, offering a faster and personalized method to combat microbial infections, and represent a promising discovery in venom biotechnology research.

## Highlights

• Two peptides designed from Natterins were subjected to *in vitro* and *in vivo* analysis.
• The peptides demonstrated antimicrobial efficacy and low cytotoxicity.
• Potential activity at specific stages of viral replication were observed.
• This approach can aid a faster and personalized method to combat microbial infections.

## 1 Introduction

Animal venoms harbor a complex blend of salts, amino acids, biogenic amines, neurotransmitters, peptides, and proteins, strategically targeting various receptors crucial for the survival of venomous creatures [1]. These venoms, along with their toxins, exhibit diverse pharmacological properties, serving as valuable resources for investigating cellular and molecular functions. Certain venom components are pivotal in human ailment and have inspired the development of novel therapeutic interventions [2]. Toxins derived from aquatic venomous organisms represent a valuable reservoir of natural compounds for both academic inquiry and practical applications. However, challenges persist in the acquisition and preservation of venom extracts, leading to the underutilization of aquatic animal venoms, especially those from fish species, as a largely untapped wellspring of novel medicines and pharmacological compounds [3,4].

Peptides, for example, are molecules found in living organisms and play a crucial role in many biological processes [[5], [6], [7], [8]]. Their widespread occurrence and functional versatility enhance their therapeutic promise [[9], [10], [11]]. Peptides are increasingly dubbed the "Goldilocks" chemical modality, characterized by their intermediate size, which combines the advantageous features of small molecules and biologics. This includes high target specificity, minimal off-target effects, and distinctive pharmacokinetic profiles [12]. Nevertheless, while a select few peptides have secured FDA approvals and others are in various stages of clinical trials [13,14], it's noteworthy that peptides have a longstanding legacy of contributing to human health spanning over a century. From insulin to vasopressin, and more recently, tirzepatide, peptides have played pivotal roles in healthcare [13,15]. In the last years, sales of peptide drugs exceeded $70 billion, with 10 non-insulin peptide drugs among the top 200 best-selling drugs, representing a substantial portion of the pharmaceutical market [16].

Peptides sourced from venom have been investigated for their potential in biotechnological applications [1,17]. While the majority of these peptides stem from a restricted range of venomous terrestrial animal groups, bioactive compounds from fish venoms have also been successfully identified and studied [18]. The therapeutic potetinal of venom/toxin-derived peptides is apparent, attributed to their heightened specificity, stability, and comprehensive evaluation of pharmacokinetic characteristics [19].

These peptides also demonstrate therapeutic attributes, notably antimicrobial properties (AMPs) [20,21]. The efficacy showcased by these substances is associated with their physicochemical characteristics, such as net charge, hydrophobicity, and solvent accessibility. These properties, in turn, govern their mechanisms of action, selectivity, and specificity towards their targets [22]. AMPs are peptides that exert their main effects on membranes, primarily by disrupting the integrity of the plasma membrane of their cellular targets [23,24]. The ability of AMPs to penetrate cells appears to bolster their antimicrobial effectiveness by engaging and disrupting intracellular components such as macromolecules and organelles [25]. However, this cellular uptake and permeability of AMPs may be linked to varying degrees of cytotoxicity. Through the design and synthesis of AMPs, it is possible to create peptides with finely tuned membrane translocation and cellular uptake abilities, coupled with reduced or minimal adverse impacts on membrane stability and cellular health. This is demonstrated by examples like buforin II, derived from the stomach tissue of the Asian toad *Bufo bufo garagrizans*, and its derivatives [26], as well as other AMPs [27].

While numerous venoms harbor AMPs, it's worth noting that other families of biologically active peptides can also be present, including cell-penetrating peptides (CPPs) [28]. CPPs comprise short sequences typically consisting of a few amino acids up to <40 residues. These peptides possess physicochemical and biological characteristics enabling them to traverse cell lipid membranes and facilitate intracellular transportation of various molecular cargoes. This transport can occur in the form of covalent conjugates or noncovalent complexes [29]. Remarkably, many of the structural and physicochemical traits found in AMPs are also present in CPPs [30,31]. Moreover, both types of peptides predominantly act on the cell membrane, inducing pore formation through diverse mechanisms that ultimately result in cellular apoptosis. These attributes not only enable their application as independent treatments but also facilitate the investigation of synergistic effects with various approved medications. These peptides can serve as adjuvants for compounds targeting the intracellular milieu, aiding in their effective delivery to the active site and bolstering treatment efficacy by combating infection or disease through diverse mechanisms of action [32,33].

Undoubtedly, the advancement of novel antimicrobial peptides (AMPs) and cell-penetrating peptides (CPPs) represents a highly promising frontier in biotechnology and therapeutic pharmacology, particularly amidst the rise of strains resistant to conventional antibiotics [34]. Nevertheless, there are notable constraints concerning the commercialization of AMPs and CPPs. These include elevated production expenses and time-intensive procedures, especially in the context of recombinant techniques, limited efficacy in animal models, heightened vulnerability to protease degradation, and diminished activity in specific physiological environments [35]. All these shortcomings can be addressed through the application of *in silico* methods to assist the design of AMPs and CPPs, termed *in silico* study (Brogden and Brogden, 2018; [36,37]).

*In silico* studies represent a logical progression adjuvant to *in vitro* methods, whereby biological and physiological processes are simulated using computer models. This approach enables researchers to explore a virtually limitless array of parameters, providing more insights or predictions regarding potential outcomes [38]. There is a growing body of literature documenting the utility of *in silico* studies in predicting, designing, and modifying AMPs and CPPs, underscoring their promise as valuable approaches [39]. Recent progress in analytical methodologies, such as the integration of genomics, mass spectrometry, and proteomics, has greatly facilitated scientists' exploration of venom compositions [40]. Coupled with contemporary high-throughput screening techniques for venom compounds, the ability to predict novel molecules encoded within toxins marks a significant advancement toward harnessing the complete therapeutic capacity of animal venoms. Leveraging high-performance technologies, it is now feasible to anticipate new molecules derived from toxins, thereby tapping into the therapeutic potential inherent in these molecules.

Moreover, biologically active peptides sourced from venomous animals indigenous to South America demonstrate significant and varied activities, presenting promising prospects as clinical candidates [41]. One such example is the TnP family of synthetic cyclic peptides discovered in the venom of *Thalassophryne nattereri*, a venomous fish inhabiting the northern and northeastern coastlines of Brazil [[42], [43], [44]].

In a previous study, our group employed *in silico* techniques to design 57 peptides derived from the *T. nattereri* family of toxins, known as Natterins [45]. Natterins have been identified as the primary agents responsible for the major toxic effects induced by *T. nattereri* venom, including local edema, and intense pain progressing to necrosis [[46], [47], [48]]. The predicted peptides exhibit a molecular mass ranging from 965.08 Da to 2704.06 Da, a net charge spanning from -2 to +7, a hydrophobic moment (μH) varying from 0.044 to 0.627, and a hydrophobic ratio ranging from 9 to 50 %. These characteristics facilitate their interaction with the microorganism membrane through adoption of an α-helix formation, ultimately leading to membrane disruption.

The present study explores the antimicrobial and antiviral properties of two selected peptides identified as promising. Upon analyzing the findings outlined in De Cena et al. [45], peptides NATT2_06 and NATT4_01 emerged as particularly noteworthy candidates with potential antimicrobial and antiviral attributes. Among the 57 peptides delineated in the investigation [45], these specific peptides showcased physicochemical characteristics deemed vital in antimicrobial and antiviral peptides as documented in existing literature. These characteristics include optimal membrane-binding potential, cellular localization both inside and outside the membrane, minimal toxicity and allergenicity, alongside ADMET parameters falling within the expected range for such molecules. Our study demonstrates that these peptides exhibited mild inhibitory effects on the growth of both Gram-positive and Gram-negative bacteria, as well as fungi, over a brief period. They demonstrated comparable inhibitory actions concerning viral replication both intra and extracellularly, without manifesting any toxic effects *in vitro* or *in vivo*. Lastly, stability and membrane interaction assessments were conducted to pave the way for these peptides to emerge as potential prototype compounds.

## 2 Materials and methods

### 2.1 Peptide design and synthesis

The peptides were designed by using a template and physicochemical base method as described in Conceição et al., [49] and de Cena et al., [45]. The NATT peptides amino acid sequence was derived from Natterins toxins from *Thalassophryne nattereri* and was used as a template protein. The peptide physicochemical properties were calculated through various tools, including ProtParam (http://web.expasy.org/protparam) [50], PepCalc (https://pepcalc.com/), Heliquest version 2 (https://heliquest.ipmc.cnrs.fr/cgi-bin/ComputParams.py) [51], and APD3 for complementary properties (https://aps.unmc.edu/prediction/predict). All mentioned software was used with its default parameter configuration.

The synthesis of NATT peptides was performed manually on solid phase following a 9 fluorenylmethoxycarbonyl (Fmoc)/*tert-butyl* (*t*-Bu) protocol [52], in polypropylene syringes fitted with a polyethylene porous disk. Solvents and soluble reagents were removed in vacuum. Commercially available reagents were used throughout without purification. Fmoc-Rink-MBHA resin (0.71 mmol/g) was used as solid support since it provides C-terminal peptides amides. Fmoc group removal was achieved with piperidine-DMF (3:7, 2 + 10 min). Coupling of commercial Fmoc-amino acids (4 or 3 equiv) were performed using DIC (4 or 3 equiv) and Oxima (4 or 3 equiv) in DMF under stirring at room temperature for 4 or 8 h The completion of the reactions was monitored by the Kaiser test [53] for amino acid bearing a primary amine and by Chloramil test [54] for the proline residue bearing a secondary amine. For each coupling and deprotection step, the resin was washed with DMF (6 × 1 min) and CH_2_Cl_2_ (3 × 1 min) and aired-dried. After coupling of ninth amino acid residue, NMP was used instead DMF. Peptide elongation was performed by repeated cycles of Fmoc removal, coupling and washings. Once the synthesis was completed, peptidyl resins were subjected to the N-terminal Fmoc removal. Then, the peptides were cleaved by treatment with TFA-H_2_O-TIS (95:2.5:2.5) for 2 h Following TFA evaporation and diethyl ether extraction, the crude peptides were purified by reverse-phase column chromatography, lyophilized, analyzed by HPLC, and characterized by high resolution mass spectrometry (HRMS) and proton nuclear magnetic resonance (1H-NMR) (Supplementary material).

### 2.2 Peptide stability analysis

Peptide stability was evaluated under various conditions. To investigate the impact of temperature on peptide structure, samples were incubated at 37 °C and 60 °C for 24 h. A control sample was kept at -4 °C. To evaluate the distribution under acidic conditions, the peptides were dissolved in a 0.074 M HCl solution, resulting in a pH of 3. For evaluation under basic conditions, the peptides were dissolved in a 0.18 M NaOH solution, resulting in a pH of 11. A control sample was maintained at pH 7. Additionally, samples were exposed to trypsin solutions at a concentration of 20 µg/mL. All samples underwent analysis using High-Performance Liquid Chromatography (HPLC) coupled with Mass Spectrometry (LC/MS-2020 EV - Prominence; Shimadzu Corporation, Tokyo, Japan). The HPLC system comprised LC-10AD mobile phase pumps, an Ultrasphere C-18 column (5 µm; 4.6 × 250 mm), a UV-vis SPD-10AV detector set at a wavelength of 220 nm, and a mass spectrometer operating in electrospray ionization (ESI) mode with a quadrupole separator.

### 2.3 Antimicrobial assay

Reference strains of *Pseudomonas aeruginosa* (ATCC 15,442), *Staphylococcus aureus* (ATCC 6538) and the fungus *Candida auris* (CBS10913 - provided by the Laboratory of Microbiology and Immunology at UNESP São José dos Campos) were evaluated in this study. All strains were stored in 20 % (v/v) glycerol at -80 °C. Before the assays, cells were seeded on Cetrimide agar for *P. aeruginosa*, agar Cled for *S. aureus* and Sabouraud dextrose agar for *C. auris*, and grown at 37 °C for 48 h The assessment of antimicrobial activity of the synthesized peptides was conducted following the analytical parameters outlined in the CLSI and NCCLS methods.

Strains were cultured in appropriate media (Mueller Hinton Broth for bacteria and Brain Heart Infusion for fungi) for approximately 24 h at 37 °C. The inoculum was adjusted to a concentration of 10^3^ cells/mL at different absorbances (630 nm for *P. aeruginosa* and *S. aureus*, and 530 nm for *C. auris*). Different concentrations of peptides were tested in sterile 96-well plates, with each well containing 200 µL (10 µL of peptide and 190 µL of inoculum in culture medium). Plates were incubated at 37 °C in a bacteriological incubator. Absorbance readings were taken by spectrophotometry at the respective wavelengths using a microplate reader (Synergy H1, BIOTEK, USA) after 2, 4, 6, 12, and 24 h. Tetracycline at 1 mg/mL was used as a positive control for bacteria, and the respective inoculum of each microorganism was used as a negative control.

After assessing biomass to determine the number of viable cells following the time-kill assay and biomass evaluation at different time points, this was subjected to Colony-Forming Unit (CFU) assessment. The CFU counting assays were performed following the method described in Herigstad [55]. This method involved the removal of 20 µL aliquots from the samples in the wells, followed by serial dilution in 180 µL of 0.9 % saline solution and plating on agar plates, which were then incubated for 24 h. After this period, the number of colonies on the plates was counted, and the number of cells per mL (CFU/mL) in the original culture was calculated.

### 2.4 Cytotoxicity assay

Murine fibroblast cells (L929) viability was assessed in the presence of the peptides using the cytotoxicity assay [56]. L929 cells were prepared in RPMI medium for the assay. After 24 h, 10 µL of each peptide concentration (3.125 µM, 6.25 µM, 12.5 µM, 25 µM, and 50 µM) were added to a 96-well plate with cells (190 µL) and incubated for 24 h at 37 °C and 5 % CO_2_. Next, 200 µL from each well was removed, and 100 µL of 3-[4,5-dimethylthiazol-2-yl]−2,5-diphenyltetrazolium bromide (MTT) 0.5 mg/mL was added to the wells. The plate was incubated for 3 h under the same conditions. Then, 100 µL from each well was removed, and 100 µL of DMSO (dimethyl sulfoxide) was added as a positive control, and PBS was added as a negative control. The multi-well plate was quantified by absorbance at 540 nm using a spectrophotometer (Synergy H1, BIOTEK, USA).

To perform the hemolytic test, human RBC was obtained from a volunteer donor. After centrifugation at 1000 xg for 5 min, the RBC pellets were resuspended in 5 % (vol/vol) sterile saline at different concentrations of peptides NATT2_06 and NATT4_01, and subsequently incubated at 37 °C for 1 hour. The supernatants were transferred to a 96-well plate, and the absorbance at 570 nm (A570) was measured. 0.12 % DMSO and 1 % TritonX-100 were used as negative and positive control, respectively [57]. The hemolysis rate was calculated as Eq 1:

$$Hemolysis\%=A_{\left(sample\right)}-A_{\left(DMSO\right)}/A_{\left(Triton\right)}-A_{\left(DMSO\right)}x100$$

This study was approved by Plataforma Brasil through the number 71282023.5.0000.5505.

### 2.5 Antiviral assays

#### 2.5.1 Viral propagation and titration of the stock

A low-passage stock of the Chikungunya virus (CHIKV, ECSA genotype, GenBank reference: KP164569) was also employed for antiviral activity. First, the viral propagation and titration were performed in the adherent African green monkey kidney epithelial (Vero) cells (ATCC CRL-1586). The virus was grown in Vero cultured in MEM medium (Gibco, Waltham, MA, USA) supplemented with 10 % fetal bovine serum (FBS, Gibco), 100 U/mL penicillin, and 100 μg/mL streptomycin (Gibco, Waltham, MA, USA)-M10) for 48 h Then, the supernatant was collected, harvested, and titrated as previously described [58]. The titer obtained for CHIKV was 9.3 × 10^6^ PFU/ml.

#### 2.5.2 Antiviral activity of peptides

The immortalized human hepatocytes derived from the hepatocarcinoma of a 57-year-old Japanese individual (Huh-7 lineage, CLS 300156) were cultured. The Huh-7 were grown in Minimum Essential Medium (Advanced MEM, Gibco®, USA), supplemented with 10 % (v/v) heat-inactivated fetal bovine serum (FBS) (Gibco®, USA), 100 U/mL penicillin, and 100 μg/mL streptomycin (PenStrep – Gibco®, USA), along with 2 mM L-glutamine (Gibco®, USA), and maintained in a humidified atmosphere with 5 % CO_2_ at 37 °C.

To analyze the antiviral potential of peptides, Huh-7 cells were seeded in 48-well flat-bottom plates (Sarstedt®, Germany) at a density of 6.5 × 10^4^ cells/well in MEM supplemented with 10 % FBS, 100 U/mL penicillin, 100 μg/mL streptomycin, and 2 mM L-glutamine. Cells were incubated for 12 h at 37 °C to allow cell adhesion to the wells. After this period, two types of assays were conducted, considering the peptides' ability to cross the plasma membrane, as described in de Cena et al., [45] and based on a previous study [59].

The first assay, named "post-treatment" (Figure S1a), involved removing the culture medium from the wells, washing with phosphate-buffered saline (PBS, pH 7.0), followed by CHIKV inoculation in MEM supplemented with 2 % FBS at a multiplicity of infection (MOI) of 1 for 2 h to allow homogeneous viral adsorption. The inoculum was then removed, and the peptides were added in triplicate at different concentrations (50, 25, 12.5, 6.25, 3.125, and 1.5625 μM, respectively), serially diluted at a 1:2 ratio in culture medium with the same supplementation mentioned. Cells were monitored for 12 h.

**Fig. 1:**
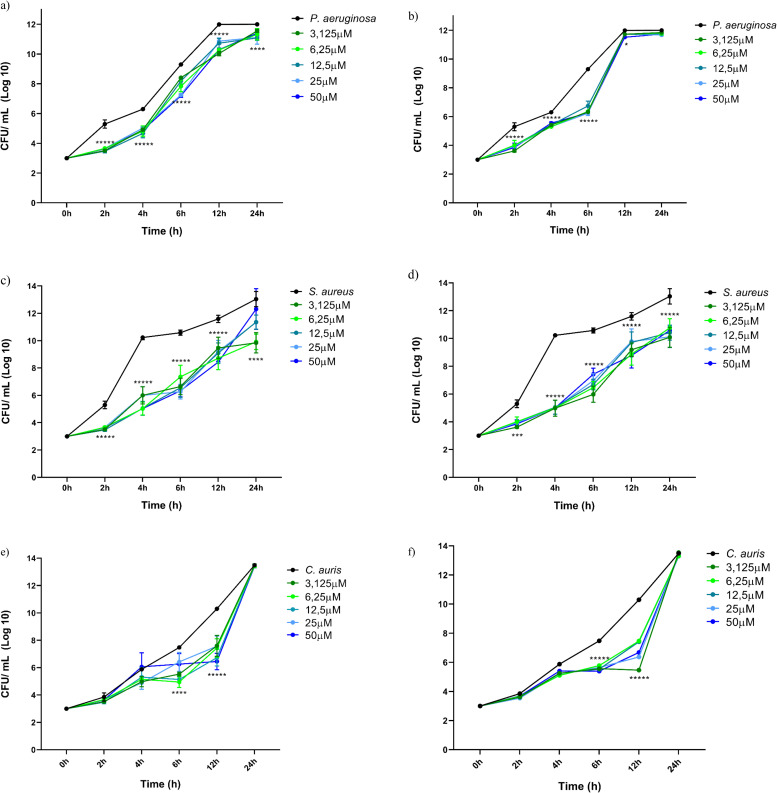
CFU/mL (Log10) of different peptides. (a) NATT2_06 against *P. aeruginosa*, (b) NATT4_01 against *P. aeruginosa*, (c) NATT2_06 against *S. aureus*, (d) NATT4_01 against *S. aureus*, (e) NATT2_06 against *C. auris*, (f) NATT4_01 against *C. auris* tested at 0, 2, 4, 6, 12 and 24 h. Statistical significance was calculated using two-way analysis of variance (ANOVA) in Prism version 8.0 (GraphPad, USA) and represented as *****p < 0.0001 for all tested concentrations, ****p < 0.0001 for four tested concentrations, ***p < 0.0001 for three tested concentrations, and *p < 0.0001 for only one tested concentration.

The second assay, named "co-treatment" (Figure S1b), involved removing the culture medium from the wells, washing with PBS, followed by simultaneous inoculation of CHIKV and peptides, diluted in the same manner in culture medium with the same supplementation, in triplicate. The same multiplicity of infection (MOI) of 1 for 2 h allows homogeneous viral adsorption. Cells were also monitored for 12 h. In all situations, positive controls were included with only CHIKV inoculation without peptide addition, and negative controls were included without CHIKV or peptide addition. At the end of each period, supernatants from each well were collected in a viral lysis buffer (AVL) and stored at -80 °C until viral RNA extraction and molecular characterization (Fig. 1(C)). The flowchart below outlines the step-by-step methodology applied in the two assays. All viral manipulation procedures were conducted in accordance with WHO and PAHO regulations in a suitable Biosafety Level 2 (BSL-2) laboratory, following the biosafety guidelines of ANVISA.

After conducting the assays, each viral RNA in the samples' supernatant was extracted using the QIAamp® Viral RNA Mini Kit (QIAGEN®, Germany). In this process, each sample underwent the extraction and purification of viral genetic material (+ssRNA) using extraction columns, with resuspension in nuclease-free ultrapure water using materials and reagents provided by the manufacturer. For the molecular characterization of NATT peptide treatment, RNA samples extracted from the plaque challenge assays' supernatants and viral stock dilutions underwent quantitative reverse transcription PCR (RT-qPCR) assays. CHIKV Primers and specific probe were synthesized by Sigma Life Science®, with 5-carboxyfluorescein (5-FAM) as the fluorophore and Minor Groove Binder (MGB-NFQ) as the fluorescence quencher [60].

For the RT-qPCR reaction, the AgPath-ID® One Step RT-qPCR kit (Applied Biosystems®, USA) was used for each extracted RNA in duplicate. The reverse transcription reaction was carried out at 45 °C for 10 min, followed by 40 amplification cycles at 95 °C for 15 s and 60 °C for 45 s on the PCR StepOne Plus® thermocycler (Applied Biosystems®, USA). Data analysis was performed using StepOne® software, version 2.3 (Applied Biosystems®, USA). C_T_ (Cycle Threshold) values were established for each sample based on the threshold automatically set by the software. All detection and quantification of viral RNA was done by real-time PCR of each sample (Table S1 and Figure S14). All the results of cycle threshold (Ct) were compared to a standard curve, which was obtained by carrying out serial dilutions from the pure stock (PFU), as previously described [61].

#### 2.6 Toxicity in *Galleria mellonella*

For this study, the methodologies described by Mylonakis et al. [62] and Jorjão et al. [63], with some modifications were employed. *G. mellonella* larvae in their final larval stage were used for the experiment. Ten randomly selected *G. mellonella* larvae of similar weight and size (250 to 350 mg) were used per group in all assays. Syringes (Hamilton Inc., USA) used for injections were sterilized with peracetic acid (Henkel - Ecolab GmbH, Düsseldorf, Germany) according to the manufacturer's instructions prior to inoculation. Each larva was injected with 10 µL of each peptide (NATT2_06 and NATT4_01) at a concentration of 50 µM into the last left proleg. A control group was injected with PBS to assess overall viability. The number of deceased *G. mellonella* was recorded every 24 h after peptide injection, with monitoring continuing for 7 days across three independent experiments. Larvae were considered dead if they showed no movement upon touch. The experiment concluded either when all larvae in the experimental group had died or transitioned into the pupal form [64].

#### 2.7 Preparation of LUVs

Large unilamellar vesicles (LUVs) of POPC:POPG (1:1), POPC:POPG (2:1) and POPC:POPG: Chol (2:1:1) with 100 nm of diameter were used as membrane model systems. The lipids were solubilized in chloroform in a round-bottom flask, and the organic solvent was evaporated under a gentle nitrogen flow to form a thin lipidic film. This film was then placed under vacuum overnight. The lipidic film was rehydrated with 10 mM phosphate buffer, pH 7.4, followed by ten freeze/thaw cycles to produce a suspension of multilamellar vesicles. Large unilamellar vesicles (LUVs) were obtained by extruding the multilamellar vesicles through 100 nm pore-size polycarbonate filters [65].

#### 2.8 Circular dichroism studies

Circular dichroism spectra of 50 µM of the peptides in 10 mM HEPES buffer, 50 mM NaF, pH 7.4, in the absence or presence of large unilamellar vesicles (LUVs), were acquired at 25 °C in the 190–260 nm wavelength range using 0.1 cm quartz cells in a JASCO model J-815 spectropolarimeter (Tokyo, Japan). Each final spectrum corresponded to an average of five scans, which were subsequently corrected for buffer or LUV baseline.

#### 2.9 Measurement of zeta (ζ) potential of LUVs in the presence of peptides

Zeta potential was measured to evaluate changes in the surface charge of the LUVs (POPC:POPG (1:1), POPC:POPG (2:1) and POPC:POPG: Chol (2:1:1)) in the presence of NATT2_06 and NATT4_01. Assays were performed in a Zetasizer Nano ZS (Malvern Instruments, Malvern, UK) equipped with a 633 nm HeNe laser and disposable ζ cells with gold electrodes. LUVs suspensions were fixed at 200 mM prepared in Mueller-Hinton broth (MHB) to a final concentration of 1 × 10^8^ CFU/mL. Each peptide solution from 0 to 30 uM, was added to the LUVs solution. Peptide-treated bacterial suspensions were dispensed into ζ cells and allowed to equilibrate for 15 min at 25 °C. The suspensions were mixed with peptides for 30 min at 37 °C. Values of viscosity and refractive index were set at 0.8872 cP and 1.330, respectively. The electrophoretic mobility of each sample was calculated, and the ζ potential was measured using the Smoluchowski equation, as previously described [66].

## 3 Results and discussion

Various studies have evaluated the prediction of antimicrobial and cell-penetrating peptides [[49], [67], [68], [69]]. Together, these studies contribute to the realm of peptide prediction and classification, offering promising applications in drug development and delivery. A prior study utilized *in silico* analysis to predict and characterize novel AMPs and CPPs derived from natterins of *T. nattereri* [45]. Subsequently, the current work evaluated the activity of two selected peptides through *in vitro* and *in vivo* assays.

The purify of chemically synthesized peptides was confirmed through High Performance Liquid Chromatography (HPLC) and High Resolution Mass Spectrometry (HRMS) (Figures s 2–7). Peptides synthesized to a purity of over 99 %, appearing as white powder, were utilized for *in vitro* and *in vivo* activity assessment. Table 1

**Table 1:** Properties of the peptides.

Peptide	SEquence	MW (g/ mol)	Charge (at pH = 7)	Theoretical PI	Boman index
NATT2_06	TTLRPKLKSK	1171.45	+4	11.26	3.02
NATT4_01	LYVAKNKYGLGKL	1466.79	+3	9.83	0.08

### 3.1 Analysis of peptide stability

Following exposure to various temperature and pH conditions, as well as treatment with Trypsin protease, peptides at a concentration of 100 μg/mL underwent analysis using high-performance liquid chromatography coupled with mass spectrometry (LC/MS-2020 EV - Prominence; Shimadzu Corporation, Tokyo, Japan). Upon subjecting the peptides to a thermal treatment of 60 °C for 24 h, no degradation peaks indicative of peptide breakdown was detected. Consequently, it can be inferred that the peptides remained stable at 37 °C, given their resilience to degradation at 60 °C. The [*M* + *H*]^+1^ values observed were 1170, 1197, and 1465, respectively, compared to the control at -4 °C, as shown in Figures S8 and S9.

Based on the HPLC chromatograms and mass spectra analysis, all three tested peptides displayed resistance to temperatures up to 60 °C. Previous research has consistently demonstrated the robust stability of antimicrobial peptides (AMPs), even at extreme temperatures such as 121 °C. For instance, Baindara et al. [70] noted that the antimicrobial activity of Penisin peptide remained intact when incubated at temperatures up to 100 °C for 30 min, albeit showing a notable decline at 121 °C. Similarly, peptides including HKPLP, Cap18, Cap11, Cap11–1-18m², Cecropin B, Cecropin P1, Melittin, Indolicidin, and Sub5 have exhibited thermal resilience, withstanding temperatures of up to 100 °C in various assays [71,72]. Additionally, Georgalaki et al. [73] reported the remarkable thermal stability of the food-grade antibiotic macedocin, which retained its activity even after short-term heating, long-term incubation for up to four weeks at 30 °C and autoclaving at 121 °C for 20 min [73].

The peptides underwent exposure to diverse pH environments, spanning from 3, 7, to 11. Under pH fluctuations between 3 and 7, none of the three tested peptides exhibited notable structural changes, suggesting their stability at pH 3. However, at pH 11, all three peptides experienced structural degradation, evidenced by distinct mass peaks observed in Figure S9e, differing from those in Figure S10c and d, corresponding to pH 3 and 7, respectively.

In mass spectrometry assays, an alkaline pH can lead to diverse outcomes. Specifically, in the analysis of proteins and peptides, an alkaline environment may induce unintended chemical modifications in certain samples, impacting ionization, fragmentation, and the detection of reference molecules [74]. Additionally, the peptides underwent treatment with a trypsin solution at a concentration of 20 μg/mL. Among them, the peptide NATT2_06 displayed notable changes in mass, as evidenced by both HPLC and MS, as depicted in Figure S12.

Regarding the assessment of the NATT4_01 peptide, there were no notable shifts observed in the chromatograms, as illustrated in Figure s13. Nonetheless, upon examination of the mass spectra, fragments of the peptide were discernible. This observation suggests potential cleavage occurring between the Lys (K) and Arg (R) residues within the peptide sequence (LYVAKNKYGLGKL).

The overall stability in plasma of NATT2_06 and NATT4_01 can be estimated using the Cavaco et al. equation that relates half-life (t_1/2_) in serum with sequence-related physicochemical properties [75]. Worthy of note, t_1/2_ of NATT2_06 is 9.3 and 90.2 min for NATT4_01, a 10-fold increase, which is in line with our finding (Figures S10 and S11).

Prior research indicates that when AMPs such as Cap18, Cecropin P1, Cecropin B, Melittin, and Indolicidin are incubated with trypsin, their antimicrobial activity is entirely lost within just 30 s. In the case of the peptide Cap11, this loss of activity occurs after 15 min of incubation. Conversely, a brief exposure of up to 5 min to trypsin enhances the antimicrobial activity of peptides such as Cap11–1-18m² by a factor of 2 [76]. Furthermore, investigations involving the peptide Penisin revealed no decline in its inhibitory activity following a 6-hour incubation with trypsin [70]. The specificity of trypsin in cleaving peptide bonds stems from its active site, which comprises specific amino acid residues (Lys and Arg) that selectively interact with particular amino acid residues within the polypeptide chain. This specificity grants trypsin the capability to identify and cleave peptide bonds at precise locations within the protein molecule. It's noteworthy that the interplay between AMPs and trypsin can be intricate and variable, suggesting the necessity for further analyzes concerning the behavior of AMPs with other proteolytic enzymes.

### 3.2 Evaluation of antimicrobial activity

The antimicrobial efficacy of the tested peptides (NATT2_06 and NATT4_01) against microorganisms was assessed by determining the colony-forming units per milliliter (UFC/mL) using the micro drop technique for cell counting [55] at time intervals of 2, 4, 6, 12, and 24 h. When evaluated against *P. aeruginosa*, peptides NATT2_06 (as shown in Fig. 1A) displayed remarkably similar activities, exhibiting significant inhibition across all tested concentrations at 2, 4, 6, and 12 h. However, at 24-hours, only the 3.1 μM concentration ceased to exhibit statistically significant inhibition, as shown in Fig. 1(A).

The peptide NATT4_01 (Fig. 1B) similarly demonstrates inhibition at all concentrations at 2, 4, and 6 h. At 12 h, it showed activity only at 50 μM, and at 24 h, no antimicrobial action was observed. A general analysis of the antimicrobial activity of the peptides reveals that they exhibit action between 2 and 12 h. The 6-hour incubation period shows the highest activity of the peptides and concentrations tested. Possibly, after this period, the peptide loses its efficacy, allowing the microorganisms to resume growth.

Liu et al. [61] successfully employed an integrated *in silico-in vitro* approach to discover bioactive peptides, marking a milestone in leveraging these methods to design molecules surpassing the potency of native peptides [61]. Given the pronounced cationicity of antimicrobial peptides (AMPs), electrostatic interactions play a pivotal role in their binding to the negatively charged cell membrane [77]. Computational analyzes have underscored that net charge and amphipathic characteristics stand out as the most statistically significant physicochemical attributes distinguishing anti-Gram-negative AMPs from others [78]. Notably, the two peptides under evaluation exhibit a positive charge of +3 and +4, respectively NATT4_01 and NATT2_06 facilitating their interaction with the negatively charged bacterial membrane. Research indicates that high cationicity in synthetically designed AMPs correlates with heightened *in vitro* antibacterial efficacy and minimal cytotoxicity, up to a threshold of +8, beyond which there's an escalation in hemolytic activity [79]. However, it's noteworthy that lower cationicity has also been associated with peptides demonstrating activity *in vivo* [80].

While the interaction between peptides and membranes is indeed a crucial aspect of AMP function, with many AMPs acting directly on microorganisms' cell membranes through affinity for certain lipid components, thereby disrupting membrane integrity and creating pores or channels, it's essential not to solely attribute their mechanism of action to electrostatic attraction or hydrophobic interactions [81]. This perspective is supported by extensive research, including studies involving the cell-penetrating peptide penetratin at 100 μM, also known as the protein transduction domain. Penetratin, a 16-residue cationic peptide (RQIKIWFQNRRMKWKK-NH_2_), derived from the third helix of the Antennapedia protein homeodomain, has been patented as a carrier peptide (or cargo transporter) for drug delivery into cells [82]. Notably, a single change of tryptophan 6 to phenylalanine in the AMP abolished its membrane transfer properties, indicating that lipid binding alone may not be sufficient for AMP activity [83]. Similarly, a W2G mutation in cecropin, an AMP predominant in insect cell-free immunity, nearly eradicated antibacterial activity [84].

There are numerous parallels between CPPs and AMPs [30]. Both types demonstrate antimicrobial effects and possess the capability to transport cargo molecules into cells. For instance, the renowned peptide LL-37 can translocate into eukaryotic cells at concentrations lower than those required for bacterial lethality, when adjusted for equivalent concentrations of divalent cations [85]. However, it's pertinent to highlight a key distinction: AMPs are perceived to possess the ability to traverse bacterial membranes autonomously, without necessitating a transport mechanism, whereas CPPs are primarily internalized via active endocytosis [86]. This discrepancy might indicate a fundamental difference in how peptides gain entry into prokaryotic versus eukaryotic cells. Among the peptides under examination, only NATT4_01 is categorized as non-CPP. However, it's noteworthy that this characteristic didn't compromise its effectiveness against *P. aeruginosa*, as it still exhibited significant activity, albeit at a lower level, following statistical analyzes.

Recently, peptides having the ability to traverse reversibly the blood-brain barrier (BBB), referred to as BBB peptide shuttles (BBBpS), were found to be a class of peptides distinct from CPP [87]. BBBpS are thus membrane-active peptides related but distinct of CPP, as CPP are related but distinct from AMP. According to the quantitative methodology adopted by Cavaco et al. [84], both NATT2_06 and NATT4_01 have moderate to high propensity (Cavaco's score function, *S* = 0.7) to traverse the BBB. This result opens an avenue for both peptides to be antimicrobial and target organs protected by physiological barriers. This is particularly relevant for NATT4_01 given its long t_1/2_.

When assessed against a strain of *S. aureus*, peptide NATT2_06 (Fig. 1c) demonstrated significant inhibition for all tested concentrations and time intervals ranging from 2 to 12 h. On the other hand, peptide NATT4_01 (illustrated in Fig. 1c) exhibited significant antimicrobial activity at all tested concentrations starting from 4 h. The peptides demonstrate antimicrobial efficacy against *S. aureus* within a time frame spanning from 4 to 12 h. Beyond this period, inhibition notably declines and ceases to maintain statistical significance, suggesting that after 12 h, the efficacy of peptide NATT2_06 diminishes, allowing bacterial growth to resume. Furthermore, it's noteworthy that the observed action does not conform to a dose-response pattern, as inhibitory activity fluctuates among the tested concentrations, and the highest concentration does not consistently correspond to the most pronounced antimicrobial effect.

*S. aureus* stands as a prime example of a Gram-positive bacterium that poses a significant global threat to human and animal health [88]. Correspondingly, findings from the current study align with those of Zhang et al. [89], who reported inhibitory effects of porcine beta defensin 2 (pBD2) against *S. aureus* within a timeframe of 1 to 8 h, demonstrating up to 80 % microbial survival inhibition within 4 h at a concentration of 150 µg/mL [89]. In comparison, the peptides examined in this study exhibited microbial survival inhibition rates ranging between 32 % and 48.8 % within the same period. Despite the peptides synthesized in this study displaying lower inhibition rates, it's noteworthy that the tested concentrations were lower, ranging from 3.16 µg/mL (NATT2_6 at 3.125 µM) to 73.3 µg/mL (NATT4_01 at 50 µM). Consequently, these findings indicate promising progress in the pursuit of potential antimicrobial agents.

In the study of Mohamed et al. [90], antimicrobial assays against various clinical and drug-resistant strains of *S. aureus* were conducted using synthetic peptides RRIKA, RR, KAF, and FAK. They found that the RRIKA peptide exhibited antimicrobial activity at concentrations ranging from 2 to 4 μM, while RR showed activity ranging from 8 to 32 μM. Conversely, the KAF and FAK peptides showed no activity against all tested strains up to 64 μM [90]. Our data reveals a significant observation: the highest concentration doesn't always result in the most favorable outcome for both AMPs and PPCs. In both studies, the concentration range showing substantial inhibitory activity was between 2 and 32 μM, despite higher concentrations being evaluated.

**Fig. 2:**
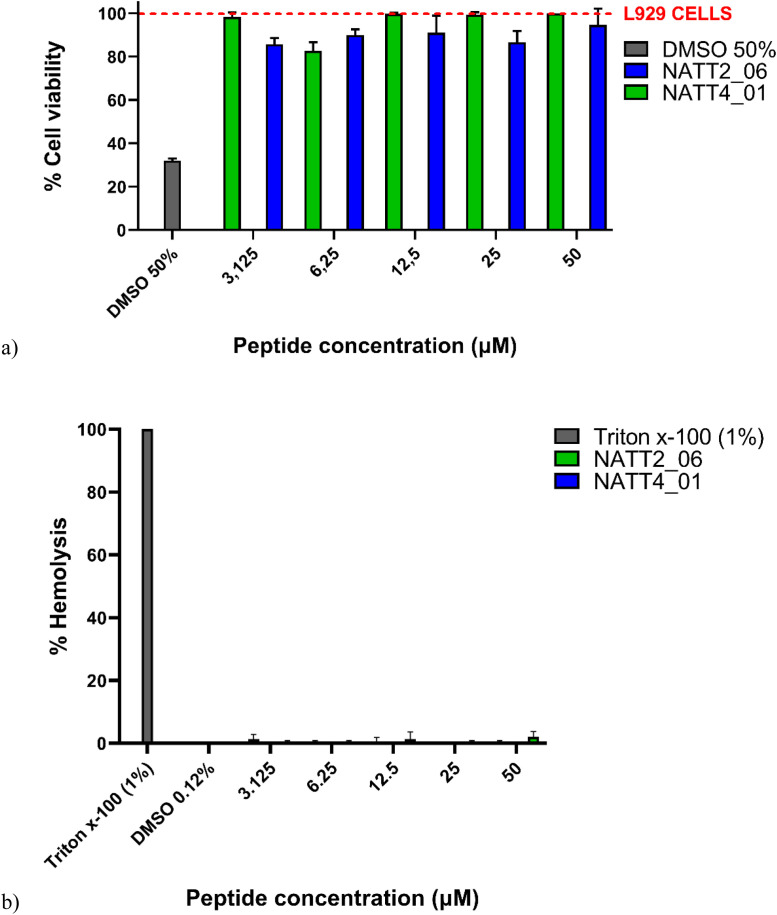
(a) Cell viability of mouse embryonic fibroblasts L929 (MEFs) treated with peptides. (b) Hemolytic activity of NATT2_06 and NATT4_01 peptides in erythrocytes. The mean standard deviation of three independent experiments is presented.

In addition to being assessed against strains of both gram-positive and gram-negative bacteria, the peptides underwent testing against a strain of the fungus *C. auris*, recognized as the most pathogenic species within its genus [91]. Upon evaluating cell viability results, as depicted in Fig. 1e and f, it becomes evident that all peptides tested exhibit a range of inhibitory effects against *C. auris* between 6 and 12 h. Conversely, peptide NATT4_01 (Fig. 2F) effectively inhibited the growth of *C. auris* at all tested concentrations at both 6 and 12 h.

AMPs known to target fungi can bind to chitin, disrupting the integrity of the fungal cell wall by increasing its permeability or forming pores [92]. Both peptides tested in our study displayed significant inhibition at least at one of the five concentrations assessed against *C. auris*, indicating potential affinity for chitin similar to the 36-amino acid peptide described by Pushpanathan et al. [92]. Many AMPs exhibit a broad spectrum of antifungal activity, proving effective against various fungal species, including drug-resistant pathogens like *Candida albicans, Aspergillus fumigatus*, and *Cryptococcus neoformans* [93].

Candidiasis represents an opportunistic infection impacting immunosuppressed and hospitalized individuals, leading to global concerns. The escalating pharmacological resistance among Candida species and the emergence of multidrug-resistant *C. auris* pose significant public health challenges [94]. AMPs have undergone extensive investigation to assess their efficacy against *C. auris*. Studies have shown that numerous AMPs exhibit noteworthy antifungal activity against *C. auris* in *in vitro* experiments. This activity encompasses the capability to impede biofilm formation and fungal growth, induce damage to the cell membrane, and facilitate fungal cell death [95].

Among the peptides evaluated, it is evident that NATT4_01 displayed superior results in inhibiting the growth of *C. auris*, spanning concentrations from 3.125 to 50 μM (equivalent to 4.58 μg/mL to 73.3 μg/mL NATT4_01). Other peptides documented in literature have also exhibited inhibitory effects against *C. auris*, including histatin-5 at 7.5 μM [96]. Histatin-5, the predominant 24-amino acid product resulting from histatin-3 cleavage, demonstrates the most potent antifungal activity among all histatins [97,98]. Moreover, recent findings indicate that the peptide LL-37 is capable of inhibiting and eradicating *C. auris* at concentrations ranging from 25 to 200 µg/mL [99]. Comparing these results with our peptides, it is evident that all fall within a similar concentration range, affirming our progress in the pursuit of effective antifungal agents.

### 3.3 Evaluation of cytotoxicity

Despite numerous efforts to develop antimicrobial peptides (AMPs) as antibiotics, one obstacle hindering the progress of many synthetic AMPs is their unknown toxicological profile upon systemic administration [100]. Recent studies have explored the toxicity of antimicrobial peptides across various cell types and organisms [43]. The results of cytotoxicity testing against L929 cells revealed that the peptides exhibited viability exceeding 75 % across all tested concentrations (ranging from 3.125 to 50 μM), as illustrated in Fig. 2a. This suggests that the peptides did not induce significant toxicity in these cells.

Hoskin and Ramamoorthy [101] investigated the toxicity of various antimicrobial peptides (AMPs) on both normal and cancer cells, underscoring the significance of determining the therapeutic index for these molecules. Moreover, the variability in the toxicity of AMPs has been documented, with certain peptides exhibiting selectivity for bacterial cells, while others may impact eukaryotic cells as well. Consequently, further research is imperative to elucidate the interplay between the structure, antimicrobial activity, and toxicity of AMPs across different cell types [101]. In this study, the structure of the tested peptides, along with their antimicrobial activity and *in vitro* and *in vivo* toxicity, were assessed. However, it's crucial to acknowledge that the observed *in vitro* toxicity effects may not necessarily mirror *in vivo* toxicity or the specific actions on target cells.

To access the hemolytic activity, the peptides NATT2_06 and NATT4_01 were incubated with the erythrocytes for 1 h at 37 °C. The highest percentage of observed hemolysis was 2 %, for NATT2_06 at 50 μM (Fig. 2b). For antimicrobial peptides to be viable for systemic applications, it's crucial for them to demonstrate low toxicity against erythrocytes [102]. The absence of hemolytic activity in the tested peptides is advantageous, as many AMPs are restricted in their use due to their significant hemolytic properties [103]. The outcomes of *in vitro* assays align with the findings proposed by De Cena et al. [45], which classified peptides NATT2_06 and NATT4_01 as unlikely to induce hemolysis based on *in silico* analyzes [45], further corroborating results obtained with L929 fibroblastic cells, indicating no adverse effects on cell growth.

In their research, Ebbensgaard et al. [76] emphasize the correlation between hydrophobicity and hemolytic activity, illustrating how the substitution of specific amino acid residues can augment peptide hemolytic activity when paired with particular amino acid residues crucial for antimicrobial efficacy (such as Leu, Ile, and Thr). Notably, both NATT2_06 and NATT4_01 peptides examined here contain Leu and Thr residues. The study indicates that merely reducing hydrophobicity or achieving a low hydrophobic moment value isn't adequate to annihilate a peptide's antimicrobial activity; instead, the amino acid composition holds significant importance [76]. The reality is that numerous AMPs possess hemolytic properties and can disrupt mammalian cells. Balancing the minimization of cellular toxicity with the maximization of antimicrobial effectiveness poses a significant challenge in the clinical application development of AMPs.

The potential for cytotoxicity is an important consideration when it comes to antimicrobial peptides. A common characteristic of positively charged AMPs is nonspecific toxicity. Most known antimicrobial peptides are cationic and cytotoxic [104].

### 3.4 Antiviral activity

The peptides NATT2_06 and NATT4_01 underwent antiviral assays to validate the findings from the *in silico* studies outlined by de Cena et al. [45]. *In vitro* assessments were conducted to evaluate the antiviral efficacy of each peptide in blocking Chikungunya virus (CHIKV) infection or progression. Two distinct assays were performed to gauge the antiviral activity at various stages of CHIKV replication. The outcome was determined based on the peptides' capacity to reduce plaque-forming units (PFU) in the supernatants of Huh-7 cell culture infected 12 h post-infection (h.p.i).

**Fig. 3:**
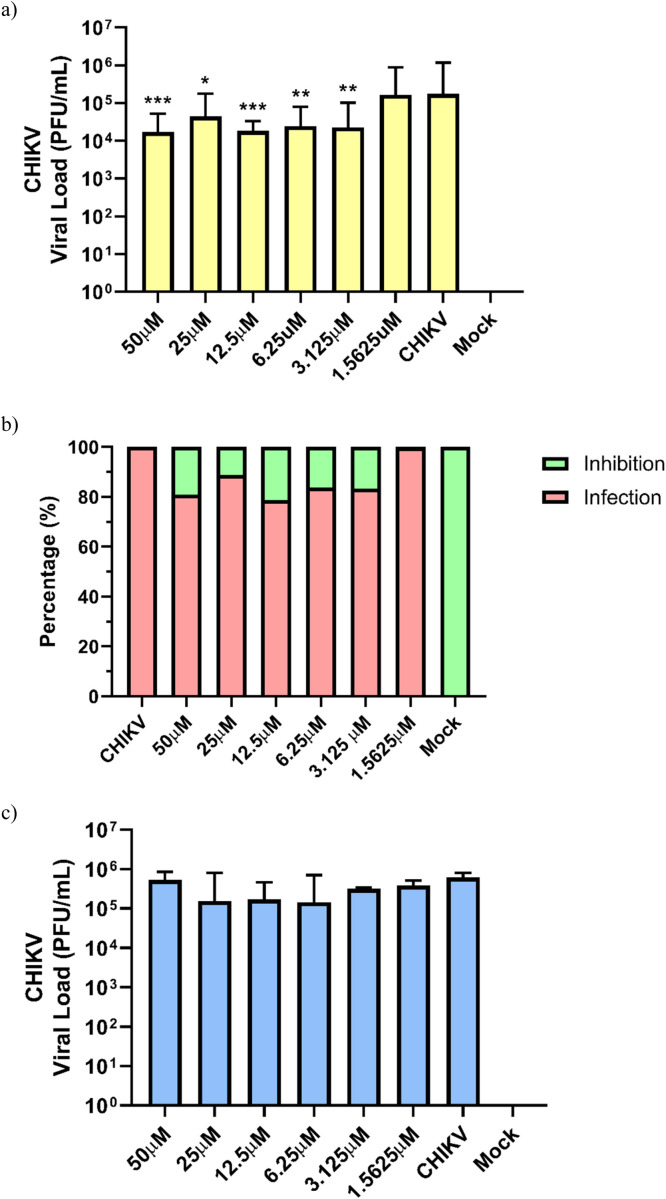
The effect on viral load reduction in post-infection treatment with peptides (a) NATT2_06 and (b) NATT4_01 during the adsorption and replication stages of CHIKV in Huh-7 cell. The p-value results (*p < 0.05, **p < 0.01, and ***p < 0.001) were calculated using Student's t-test with and without Welch's correction for samples with a normal distribution (50, 25, 12.5, 3.125, and 1.5625 μM) or the Mann-Whitney test (6.25 μM) for samples with a non-normal distribution, using the CHIKV group as a control sample. (c) Percentage of viral inhibition at different concentrations of the NATT2_06 peptide: 19.12 % (50 μM), 11.09 % (25 μM), 16.25 % (6.25 μM) and 16.63 % (3.125 μM). The green bars represent the levels of viral load inhibition in the presence of NATT2_06 in post-infection treatment of CHIKV. The green bars represent the levels of viral load inhibition in the presence of NATT2_06 post-infection CHIKV treatment. Inhibition was assessed in a dose-dependent manner. Inhibition was assessed in a dose-dependent manner.

**Fig. 4:**
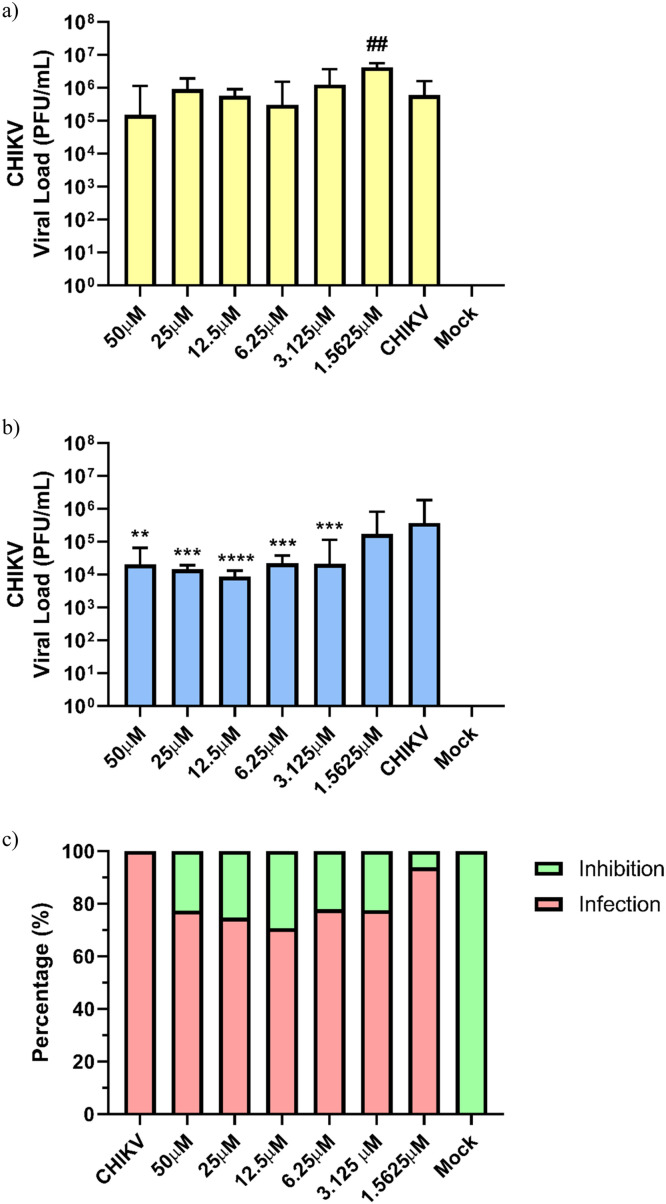
The effect on the reduction of viral load in co-treatment during CHIKV infection with the peptides (A) NATT 2_06 and (B) NATT4_01. The p-value results (*p < 0.05, **p < 0.01, ***p < 0.001, and ****p < 0.0001) were calculated using the Student's t-test with and without Welch correction at all concentrations, using the CHIKV group as the control sample. (C) Percentage of viral inhibition under different concentrations of the peptide NATT4_01: 22,62 % (50 μM), 25,31 % (25 μM), 22,08 % (6,25 μM) e 22,44 % (3125 μM). The figure shows the results of three independent experiments.

The inhibitory effects of peptides NATT2_06 and NATT4_01 on CHIKV replication were dose-dependent, with peptide concentrations ranging from 1.5625 to 50 μM. However, minimal antiviral activity against CHIKV was noted in cells treated with either NATT2_06 or NATT4_01 (Fig. 3, Fig. 4 and Table S2).

For the first assay, the treatment with peptides commenced 2 h post-viral infection, following the adsorption and entry stage of the virus into the cell. The final viral load recovered by the cells after the 12-hour assay is illustrated in Fig. 3a and b. Notably, the inhibitory effect on CHIKV replication was solely evident with the NATT2_06 peptide. At a concentration of 12.5 μM, NATT2_06 exhibited a reduction about ten times in the quantity of plaque-forming units (PFU) (4.11 ± 0.45 Log_10_ (PFU/mL), < 0.0001), corresponding to a 21.41 % inhibition of viral load compared to the untreated control (5.23 ± 0.73 Log_10_ (PFU/mL)) (Fig. 3c). The inhibitory effect of NATT2_06 treatment persisted up to a concentration of 3.125 μM (4.36 ± 0.65 Log_10_ (PFU/mL)). Conversely, no significant reduction in viral load was observed with NATT4_01 treatment in this assay, indicating no inhibition of CHIKV replication (Fig. 3b).

The studies outlined by de Cena et al. [45] categorize the NATT4_01 peptide as "Non-CPP," unlike NATT2_06, which is classified as "CPP." These classifications offer insight into the assay results. If a peptide fails to penetrate the cell, it cannot interact with the internalized virus, thereby lacking inhibitory activity, as observed with NATT4_01. In a second assay, peptide treatment was administered concurrently with viral infection. The final viral load recovered by the Huh-7 cells after the 12-hour assay is illustrated in Fig. 4a and b. Notably, antiviral activity against CHIKV was solely observed in the treatment with the NATT4_01 peptide. A reduction of about 10 times in the plaque-forming units (PFU) at a concentration of 12.5 μM (3.94 ± 0.18 Log_10_ (PFU/mL), *p* < 0.0001) was evident, with a corresponding decrease in viral load of 29.26 % (Fig. 4b), compared to the untreated control (5.57 ± 0.69 Log_10_ (PFU/mL)). This effect of NATT4_01 persisted up to the concentration of 3.125 μM (4.32 ± 0.74 Log_10_ (PFU/mL)). In this assay, no significant reduction in viral load was observed with treatments using NATT2_06, indicating no activity of the peptides on CHIKV (Fig. 4a).

Arthropod-borne viruses (arboviruses), such as the chikungunya virus (CHIKV), are the primary pathogens of interest for global public health [[105], [106], [107]]. Therefore, there is a growing need to develop new drugs to treat these viral infections. In this context, AMPs obtained from animal venoms stand out as promising compounds for exhibiting strong antiviral activity against emerging arboviral pathogens [43,108]. These peptides may have direct antiviral effects on viral particles or replication cycles or exert indirect antiviral effects by modulating the host's immune response [108].

Numerous peptides are undergoing clinical trials as potential antimicrobial agents, owing to their promising antiviral activity against specific viral pathogens and distinctive mechanisms of action. Examples include Myrcludex B, Hepalatide (L47), Adaptavir, and Aviptadil [109]. Additionally, T20© (enfuvirtide) is a peptide currently utilized in the combined therapy of HIV-1 infections, inhibiting the entry of HIV into human cells by preventing viral fusion with the cell membrane. Despite being the sole commercially available peptide for this purpose, T20© faces limitations such as a low genetic barrier to drug resistance and a short *in vivo* half-life [[110], [111], [112], [113]].

Lima et al. [43] investigated the efficacy of Latarcin 1 peptide against CHIKV at varying concentrations ranging from 0.5 to 50 μM, alongside assays involving the NATT peptides. As outlined by Rothan et al. [114], Latarcin 1 peptide exhibits multifaceted action throughout the viral replication cycle, including entry, assembly/release, fusion, and replication stages. Interestingly, Latarcin 1 demonstrated diminished inhibitory potential against CHIKV during the pre-treatment phase compared to simultaneous addition with the virus inoculum and post-infection treatment. This observation suggests that the peptide exhibits greater potential for inhibiting viral activity when administered alongside the virus or after viral infection, akin to the actions observed with NATT4_01 and NATT2_06 peptides, respectively. Hence, it is plausible that the tested peptides may exert their effects at distinct stages of viral replication.

Antiviral peptides are characterized by their cationic nature and amphipathic properties, making them promising candidates for therapeutic applications [115]. These attributes are particularly advantageous in combating enveloped arboviruses like flaviviruses and alphaviruses. The viral envelope originates from host cell membranes, comprising lipid rafts, sphingolipids, and cholesterol, thereby exhibiting an amphipathic nature and negative charge. Consequently, cationic peptides can electrostatically interact with this viral structure, leading to direct virucidal effects or interference with virus binding and fusion during the viral life cycle within host cells [116]. Moreover, they have the capability to disrupt endoplasmic reticulum membranes, thus impeding exponential virus replication [117].

The main advantages of peptides over small chemical compounds are specificity, tolerability, potency, rarer side effects (since the decomposition products are amino acids), and commercial scalability. Moreover, peptides have the potential to interact at the active site of large proteins where protein-protein interaction is essential. Identifying compounds has become much easier now with advances in structural and genomic technologies. However, short half-life, solubility, bioavailability, stability, and natural peptide delivery are the main challenges faced by these peptides [115].

### 3.5 Toxicity in *G. mellonella*

**Fig. 5:**
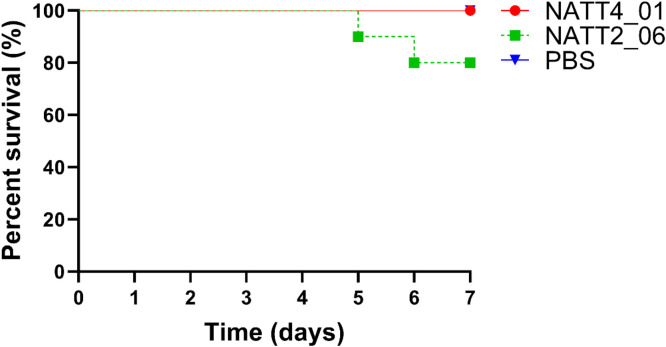
Toxicity of NATT2_06 and NATT4_01 in *G. mellonella.*

The *in vivo* toxicity of peptides (NATT2_06 and NATT4_01) was assessed using *G. mellonella* larvae (Fig. 5). The highest concentration tested in all assays was 50 µM (equivalent to 58.6 µg/larvae for NATT2_06 and 73.3 µg/larvae for NATT4_01). No significant toxicity was observed in any of the peptide samples tested. Over the course of the 7-day experiment, only three deaths occurred in the group treated with peptide NATT2_06 (on days 5, 6, and 7), while no deaths were observed in the group treated with NATT4_01.

*G. mellonella* larvae have emerged as a valuable model for assessing both the *in vivo* toxicity and efficacy of antimicrobial agents [118]. Notably, there exists a robust correlation between the toxicity of food preservatives observed in *Galleria larvae* and that in rats, underscoring the model's potential for evaluating *in vivo* toxicity of various compounds [119]. Insects possess a highly sensitive immune response, and the introduction of foreign material, such as pathogens or pathogen-associated material, can trigger a potent antimicrobial immune response within the insect, rendering it resistant to subsequent infections—a phenomenon known as priming [120]. Moreover, the simplicity and precision of inoculation and control procedures have established Galleria as the predominant model organism in larval studies [[121], [122], [123]].

### 3.6 CD and zeta potential measurements of LUVs in the presence of peptides

**Fig. 6:**
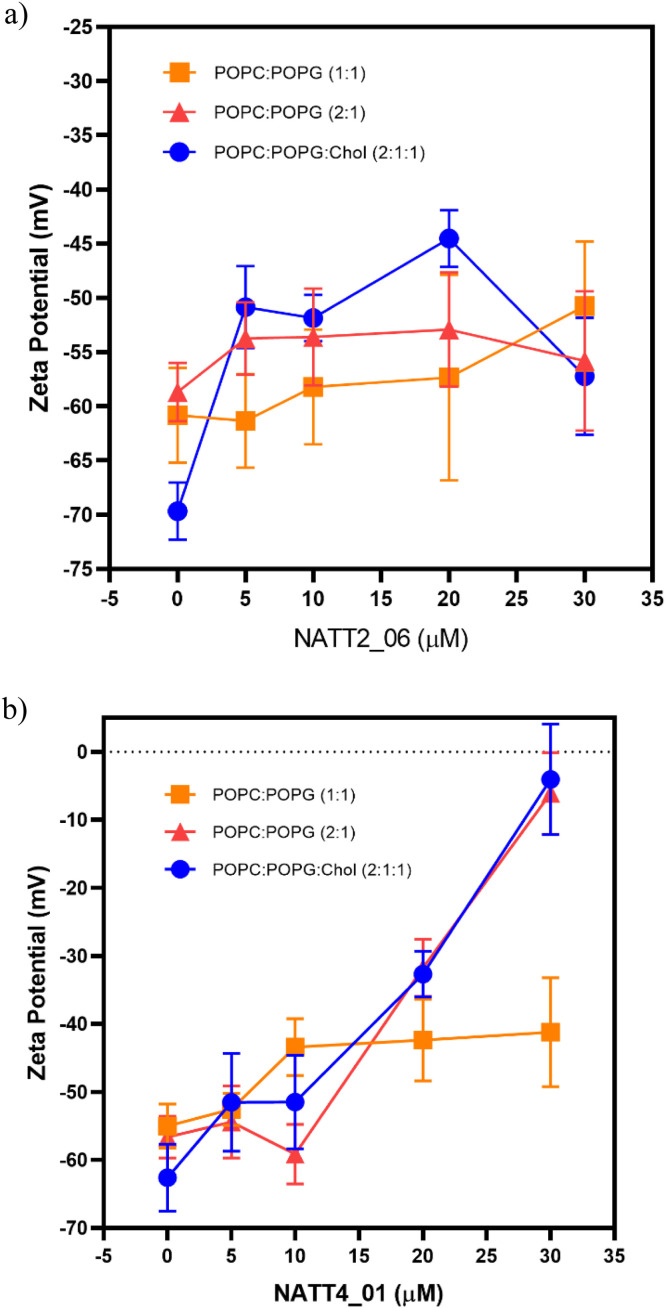
Zeta-potential for membrane model systems in the presence of (A) NATT4_01 and (B) NATT2_06. Bars represent the zeta-potential range. POPC:POPG (1:1) (orange squares); POPC:POPG (2:1) (pink triangles) and POPC:POPG:Chol (2:1:1) (blue circles). The lipid concentration was kept constant at 200 mM, while peptide concentration ranged from 0 to 30 μM.

Circular dichroism (CD) spectroscopy indicated that the secondary structure of these peptides remained unaffected upon interaction with POPC:POPG LUVs. However, the incorporation of cholesterol into the LUVs (POPC:POPG:Chol) slightly altered only the secondary structure of NATT4_01 (Figure S15). The zeta potential, derived from the mobility of cells in an electric field under defined pH and salt conditions, offers insights into cell surface charge [49]. Assessments of zeta potential using model membrane systems revealed variations across different peptides, indicating that NATT4_01 more efficiently achieves charge neutralization in both POPG:POPC (2:1) and POPC:POPG:Cholesterol (2:1:1) setups (Fig. 6b).

Moreira Brito investigated the synthetic antimicrobial peptide LyeTx Ib Cys, derived from LyeTx I found in the venom of the spider *Lycosa erythrognata*. Their study revealed that when subjected to zeta potential tests on POPC:POPG LUV membranes, it elicited an increase in the membrane's surface charge, even at relatively low concentrations ranging from 20 μM to 40 μM [124]. These concentrations mirror those used in assays with peptides NATT2_06 and NATT4_01, indicating that synthetic antimicrobial peptides indeed interact with membranes.

Another investigation into zeta potential analysis in POPC:POPG LUVs, this time utilizing the lipopeptide polymyxin B, indicates that in the presence of the peptide, the zeta potential data display a trend towards less negative values. This outcome suggests that initial electrostatic interactions play a significant role in peptide binding [125]. Despite interacting with LPS, there is not complete neutralization of the membrane, as seen in assays with peptides derived from natterins, hinting that polymyxin B may not fully access the negative charges of LPS aggregates, similar to the behavior expected from the peptides studied by our group.

Moreover, Domingues and colleagues [66] propose that most cationic peptides can prompt aggregation of negatively charged lipid vesicles at concentrations considered high. Their study also highlights that many hydrophobic peptides can interact with neutrally charged lipids and induce their aggregation, suggesting these properties hold promise in the design of new peptides with antibiotic activity [66].

## 4 Conclusion

This study investigates the intricate dynamics of antimicrobial and cell-penetrating peptides (AMPs and CPPs) derived from Natterin toxin, exploring their stability, antimicrobial efficacy, cytotoxicity, and antiviral activity. The findings underscore the peptides' robust stability under varying temperatures and pH conditions, alongside notable resistance to proteolytic degradation. The antimicrobial assays reveal significant efficacy against *P. aeruginosa, S. aureus* and *C. auris*, with varying degrees of inhibition observed across different time intervals and concentrations. Moreover, the minimal cytotoxicity and hemolytic activity demonstrated by the peptides enhance their potential as viable therapeutic agents. The antiviral assays, although revealing limited efficacy against the Chikungunya virus, highlight distinct stages of viral replication where the peptides may exert their effects. Additionally, the *in vivo* toxicity assessment using *G. mellonella* larvae provides promising indications of the peptides' safety profiles. Finally, the zeta potential measurements offer insights into the peptides' interactions with model membranes, further elucidating their potential mechanisms of action. As the landscape of antimicrobial resistance continues to evolve, the continuous exploration and refinement of AMPs and CPPs are imperative. This study not only contributes valuable data to the existing body of knowledge but also guides the way for future research endeavors aimed at harnessing the full therapeutic potential of these peptides. The journey towards effective antimicrobial and antiviral agents is arduous, yet the insights gained from this research offer a light of hope in the field of drug development and delivery.

## Ethics

The study and protocols have been approved by the ethics committee of centre (Plataforma Brasil through the number 71282023.5.0000.5505). The study is conducted according to good clinical practice and the Declaration of Helsinki.

## Funding

This work was supported by the Fundação de Amparo à Pesquisa do Estado de São Paulo (FAPESP) [2021/04316-9].

## CRediT authorship contribution statement

**Gabrielle L. de Cena:** Writing – review & editing, Writing – original draft, Investigation, Formal analysis, Data curation. **Dayane B. Tada:** Writing – original draft, Methodology, Investigation, Formal analysis. **Danilo B.M. Lucchi:** Methodology, Investigation, Formal analysis. **Tiago A.A. Santos:** Writing – original draft, Methodology, Formal analysis, Data curation. **Montserrat Heras:** Writing – review & editing, Writing – original draft, Methodology, Investigation, Formal analysis, Conceptualization. **Maria Juliano:** Methodology, Investigation, Formal analysis. **Carla Torres Braconi:** Writing – review & editing, Writing – original draft, Methodology, Investigation, Formal analysis. **Miguel A.R.B. Castanho:** Writing – review & editing, Writing – original draft, Validation, Investigation, Formal analysis, Data curation. **Mônica Lopes-Ferreira:** Writing – review & editing, Writing – original draft, Supervision, Investigation, Formal analysis, Conceptualization. **Katia Conceição:** Writing – review & editing, Writing – original draft, Supervision, Resources, Project administration, Methodology, Investigation, Formal analysis, Data curation, Conceptualization.

## Declaration of competing interest

The authors declare that they have no known competing financial interests or personal relationships that could have appeared to influence the work reported in this paper.

## Data Availability

No data was used for the research described in the article.

## Appendix Supplementary materials

Image, application 1

## References

[1] Pennington M.W., Czerwinski A., Norton R.S. (2018). Peptide therapeutics from venom: current status and potential. Bioorg. Med. Chem..

[2] Bordon K.de C.F., Cologna C.T., Fornari-Baldo E.C., Pinheiro-Júnior E.L., Cerni F.A., Amorim F.G., Anjolette F.A.P., Cordeiro F.A., Wiezel G.A., Cardoso I.A., Ferreira I.G., de Oliveira I.S., Boldrini-França J., Pucca M.B., Baldo M.A., Arantes E.C. (2020). From animal poisons and venoms to medicines: achievements, challenges and perspectives in drug discovery. Front. Pharmacol..

[3] Church J.E., Hodgson W.C. (2002). The pharmacological activity of fish venoms. Toxicon..

[4] Chekan J.R., Fallon T.R., Moore B.S. (2020). Biosynthesis of marine toxins. Curr. Opin. Chem. Biol..

[5] Padhi A., Sengupta M., Sengupta S., Roehm K.H., Sonawane A. (2014). Antimicrobial peptides and proteins in mycobacterial therapy: current status and future prospects. Tuberculosis. (Edinb).

[6] Giordano C., Marchiò M., Timofeeva E., Biagini G. (2014). Neuroactive peptides as putative mediators of antiepileptic ketogenic diets. Front. Neurol..

[7] Buchwald H., Dorman R.B., Rasmus N.F., Michalek V.N., Landvik N.M., Ikramuddin S. (2014). Effects on GLP-1, PYY, and leptin by direct stimulation of terminal ileum and cecum in humans: implications for ileal transposition. Surg. Obes. Relat. Dis..

[8] Fosgerau K., Hoffmann T. (2015). Peptide therapeutics: current status and future directions. Drug Discov. Today.

[9] Fjell C.D., Hiss J.A., Hancock R.E.W., Schneider G. (2011). Designing antimicrobial peptides: form follows function. Nat. Rev. Drug Discov..

[10] Hancock R.E.W., Sahl H.-G. (2006). Antimicrobial and host-defense peptides as new anti-infective therapeutic strategies. Nat. Biotechnol..

[11] Diochot S., Baron A., Salinas M., Douguet D., Scarzello S., Dabert-Gay A.-S., Debayle D., Friend V., Alloui A., Lazdunski M., Lingueglia E. (2012). Black mamba venom peptides target acid-sensing ion channels to abolish pain. Nature.

[12] Hickey J.L., Sindhikara D., Zultanski S.L., Schultz D.M. (2023). Beyond 20 in the 21st century: prospects and challenges of non-canonical amino acids in peptide drug discovery. ACS. Med. Chem. Lett..

[13] Usmani S.S., Bedi G., Samuel J.S., Singh S., Kalra S., Kumar P., Ahuja A.A., Sharma M., Gautam A., Raghava G.P.S. (2017). THPdb: database of FDA-approved peptide and protein therapeutics. PLoS. One.

[14] Herzig V., Cristofori-Armstrong B., Israel M.R., Nixon S.A., Vetter I., King G.F. (2020). Animal toxins - Nature's evolutionary-refined toolkit for basic research and drug discovery. Biochem. Pharmacol..

[15] Chavda V.P., Ajabiya J., Teli D., Bojarska J., Apostolopoulos V., Tirzepatide a (2022). New era of dual-targeted treatment for diabetes and obesity: a mini-review. Molecules..

[16] Wang L., Wang N., Zhang W., Cheng X., Yan Z., Shao G., Wang X., Wang R., Fu C. (2022). Therapeutic peptides: current applications and future directions. Signal. Transduct. Target. Ther..

[17] Holford N., Channon S., Heron J., Jones I. (2018). The impact of postpartum psychosis on partners. BMC. Pregnancy. ChildBirth.

[18] Ziegman R., Alewood P. (2015). Bioactive components in fish venoms. Toxins. (Basel).

[19] Lewis R.J., Garcia M.L. (2003). Therapeutic potential of venom peptides. Nat. Rev. Drug Discov..

[20] Akef H.M. (2019). Anticancer and antimicrobial activities of scorpion venoms and their peptides. Toxin. Rev..

[21] Torres M.D.T., Silva A.F., Andrade G.P., Pedron C.N., Cerchiaro G., Ribeiro A.O., Oliveira V.X., de la Fuente-Nunez C. (2020). The wasp venom antimicrobial peptide polybia-CP and its synthetic derivatives display antiplasmodial and anticancer properties. Bioeng. Transl. Med..

[22] Torres M.D.T., de la Fuente-Nunez C. (2019). Reprogramming biological peptides to combat infectious diseases. Chem. Commun..

[23] Neundorf I. (2019). Antimicrobial and cell-penetrating peptides: how to understand two distinct functions despite similar physicochemical properties. Adv. Exp. Med. Biol..

[24] Splith K., Neundorf I. (2011). Antimicrobial peptides with cell-penetrating peptide properties and vice versa. Eur. Biophys. J..

[25] Le C.-F., Fang C.-M., Sekaran S.D. (2017). Intracellular targeting mechanisms by antimicrobial peptides. Antimicrob. Agents Chemother.

[26] Park C.B., Yi K.S., Matsuzaki K., Kim M.S., Kim S.C. (2000). Structure-activity analysis of buforin II, a histone H2A-derived antimicrobial peptide: the proline hinge is responsible for the cell-penetrating ability of buforin II. Proc. Natl. Acad. Sci. U. S. A..

[27] Liang Y., Zhang X., Yuan Y., Bao Y., Xiong M. (2020). Role and modulation of the secondary structure of antimicrobial peptides to improve selectivity. Biomater. Sci..

[28] Rádis-Baptista G. (2021). Cell-penetrating peptides derived from animal venoms and toxins. Toxins. (Basel).

[29] Thomas A., Lins L., Divita G., Brasseur R. (2010). Realistic modeling approaches of structure-function properties of CPPs in non-covalent complexes. Biochim. Biophys. Acta.

[30] Henriques S.T., Melo M.N., Castanho M.A.R.B. (2006). Cell-penetrating peptides and antimicrobial peptides: how different are they?. Biochem. J..

[31] Yacoub T., Rima M., Karam M., Fajloun J.-M.S.A.Z. (2020). Antimicrobials from venomous animals: an overview. Molecules..

[32] Lamiyan A.K., Dalal R., Kumar N.R. (2020). Venom peptides in association with standard drugs: a novel strategy for combating antibiotic resistance - an overview. J. Venom. Anim. Toxins. Incl. Trop. Dis..

[33] Freire J.M., Veiga A.S., Rego de Figueiredo I., de la Torre B.G., Santos N.C., Andreu D., Da Poian A.T., Castanho M.A.R.B. (2014). Nucleic acid delivery by cell penetrating peptides derived from dengue virus capsid protein: design and mechanism of action. FEBS. J..

[34] Agrawal P., Raghava G.P.S. (2018). Prediction of antimicrobial potential of a chemically modified peptide from its tertiary structure. Front. Microbiol..

[35] Brogden N.K., Brogden K.A. (2011). Will new generations of modified antimicrobial peptides improve their potential as pharmaceuticals?. Int. J. Antimicrob. Agents.

[36] Bhadra P., Yan J., Li J., Fong S., Siu S.W.I. (2018). AmPEP: sequence-based prediction of antimicrobial peptides using distribution patterns of amino acid properties and random forest. Sci. Rep..

[37] Mandal S.M., Roy A., Ghosh A.K., Hazra T.K., Basak A., Franco O.L. (2014). Challenges and future prospects of antibiotic therapy: from peptides to phages utilization. Front. Pharmacol..

[38] Colquitt R.B., Colquhoun D.A., Thiele R.H. (2011). In silico modelling of physiologic systems. Best. Pract. Res. Clin. Anaesthesiol..

[39] Roy A., Nair S., Sen N., Soni N., Madhusudhan M.S. (2017). In silico methods for design of biological therapeutics. Methods.

[40] Slagboom J., Kaal C., Arrahman A., Vonk F.J., Somsen G.W., Calvete J.J., Wüster W., Kool J. (2022). Analytical strategies in venomics. Microchemical J..

[41] Ageitos L., Torres M.D.T., de la Fuente-Nunez C. (2022). Biologically active peptides from venoms: applications in antibiotic resistance, cancer, and beyond. Int. J. Mol. Sci..

[42] Lima C., Falcão M.A.P., Pinto F.J., Bernardo J.T.G., Lopes-Ferreira M. (2023). The anti-inflammatory peptide TnP is a candidate molecule for asthma treatment. Cells.

[43] Lima C., Eto S.F., Lopes-Ferreira M. (2022). Shedding light on the drug-target prediction of the anti-inflammatory peptide TnP with bioinformatics tools. Pharmaceuticals. (Basel).

[44] Komegae E.N., Souza T.A.M., Grund L.Z., Lima C., Lopes-Ferreira M. (2017). Multiple functional therapeutic effects of TnP: a small stable synthetic peptide derived from fish venom in a mouse model of multiple sclerosis. PLoS. One.

[45] De Cena G.L., Scavassa B.V., Conceição K. (2022). In silico prediction of anti-infective and cell-penetrating peptides from Thalassophryne nattereri Natterin toxins. Pharmaceuticals.

[46] Magalhães G.S., Junqueira-de-Azevedo I.L.M., Lopes-Ferreira M., Lorenzini D.M., Ho P.L., Moura-da-Silva A.M. (2006). Transcriptome analysis of expressed sequence tags from the venom glands of the fish Thalassophryne nattereri. Biochimie.

[47] Magalhães G.S., Lopes-Ferreira M., Junqueira-de-Azevedo I.L.M., Spencer P.J., Araújo M.S., Portaro F.C.V., Ma L., Valente R.H., Juliano L., Fox J.W., Ho P.L., Moura-da-Silva A.M. (2005). Natterins, a new class of proteins with kininogenase activity characterized from Thalassophryne nattereri fish venom. Biochimie.

[48] Lopes-Ferreira M., Emim J.A.da S., Oliveira V., Puzer L., Cezari M.H., Araújo M.da S., Juliano L., Lapa A.J., Souccar C., Moura-da-Silva A.M. (2004). Kininogenase activity of Thalassophryne nattereri fish venom. Biochem. Pharmacol..

[49] Conceição K., de Cena G.L., da Silva V.A., de Oliveira Neto X.A., de Andrade V.M., Tada D.B., Richardson M., de Andrade S.A., Dias S.A., Castanho M.A.R.B., Lopes-Ferreira M. (2020). Design of bioactive peptides derived from CART sequence isolated from the toadfish Thalassophryne nattereri. Biotech.

[50] Gasteiger E., Hoogland C., Gattiker A., Duvaud S., Wilkins M.R., Appel R.D., Bairoch A., Walker John M. (2005). The Proteomics Protocols Handbook.

[51] Gautier R., Douguet D., Antonny B., Drin G. (2008). HELIQUEST: a web server to screen sequences with specific α-helical properties. Bioinformatics..

[52] Chan W., White P. (1999). Fmoc solid phase peptide synthesis: a practical approach.

[53] Kaiser E., Colescott R.L., Bossinger C.D., Cook P.I. (1970). Color test for detection of free terminal amino groups in the solid-phase synthesis of peptides. Anal. Biochem..

[54] Vojkovsky T. (1995). Detection of secondary amines on solid phase. Pept. Res..

[55] Herigstad B., Hamilton M., Heersink J. (2001). How to optimize the drop plate method for enumerating bacteria. J. Microbiol. Methods.

[56] Riss T.L., Moravec R.A., Niles A.L., Duellman S., Benink H.A., Worzella T.J., Minor L., Markossian S., Grossman A., Arkin M., Auld D., Austin C., Baell J. (2004). Assay Guidance Manual.

[57] Conceição K., Konno K., Richardson M., Antoniazzi M.M., Jared C., Daffre S., Camargo A.C., Pimenta D.C. (2006). Isolation and biochemical characterization of peptides presenting antimicrobial activity from the skin of Phyllomedusa hypochondrialis. Peptides..

[58] J.J.H. Chu, S.K. Ang, eds., Chikungunya Virus: Methods and Protocols, Springer, New York, NY, 2016. 10.1007/978-1-4939-3618-2.

[59] Mandova T., Saivish M.V., La Serra L., Nogueira M.L., Da Costa F.B (2023). Identification of potential antiviral hops compounds against chikungunya virus. Int. J. Mol. Sci..

[60] Patel P., Abd El Wahed A., Faye O., Prüger P., Kaiser M., Thaloengsok S., Ubol S., Sakuntabhai A., Leparc-Goffart I., Hufert F.T., Sall A.A., Weidmann M., Niedrig M. (2016). A field-deployable reverse transcription recombinase polymerase amplification assay for rapid detection of the Chikungunya Virus. PLoS Negl. Trop..

[61] Liu S.-Q., Li X., Deng C.-L., Yuan Z.-M., Zhang B. (2018). Development and evaluation of one-step multiplex real-time RT-PCR assay for simultaneous detection of Zika virus and Chikungunya virus. J. Med. Virol..

[62] Mylonakis E., Moreno R., El Khoury J.B., Idnurm A., Heitman J., Calderwood S.B., Ausubel F.M., Diener A. (2005). Galleria mellonella as a model system to study Cryptococcus neoformans pathogenesis. Infect. Immun..

[63] Jorjão A.L., de Oliveira F.E., Leão M.V.P., Jorge A.O.C., de Oliveira L.D. (2018). Effect of Lactobacillus rhamnosus on the response of Galleria mellonella against Staphylococcus aureus and Escherichia coli infections. Arch. Microbiol..

[64] Dos Santos J.D., de Alvarenga J.A., Rossoni R.D., García M.T., Moraes R.M., Anbinder A.L., Cardoso Jorge A.O., Junqueira J.C. (2017). Immunomodulatory effect of photodynamic therapy in Galleria mellonella infected with Porphyromonas gingivalis. Microb. Pathog..

[65] Mendonça D.A., Figueira T.N., Melo M.N., Harder O., Niewiesk S., Moscona A., Porotto M., Veiga A.S. (2019). Self-assembly stability compromises the efficacy of tryptophan-containing designed anti-measles virus peptides. J. Nanomed. Nanotechnol..

[66] Domingues M.M., Santiago P.S., Castanho M.A.R.B., Santos N.C. (2008). What can light scattering spectroscopy do for membrane-active peptide studies?. J. Pept. Sci..

[67] Duque-Salazar G., Mendez-Otalvaro E., Ceballos-Arroyo A.M., Orduz S. (2020). Design of antimicrobial and cytolytic peptides by computational analysis of bacterial, algal, and invertebrate proteomes. Amino Acids..

[68] Beltran J.A., Del Rio G., Brizuela C.A. (2020). An automatic representation of peptides for effective antimicrobial activity classification. Comput. Struct. Biotechnol. J..

[69] Chen L., Chu C., Huang T., Kong X., Cai Y.-D. (2015). Prediction and analysis of cell-penetrating peptides using pseudo-amino acid composition and random forest models. Amino Acids..

[70] Baindara P., Chaudhry V., Mittal G., Liao L.M., Matos C.O., Khatri N., Franco O.L., Patil P.B., Korpole S. (2015). Characterization of the antimicrobial peptide penisin, a class Ia novel lantibiotic from paenibacillus sp. Strain A3. Antimicrob Agents Chemother.

[71] Ebbensgaard A., Mordhorst H., Overgaard M.T., Nielsen C.G., Aarestrup F.M., Hansen E.B. (2015). Comparative evaluation of the antimicrobial activity of different antimicrobial peptides against a range of pathogenic bacteria. PLoS. One.

[72] Sun D., Wu S., Jing C., Zhang N., Liang D., Xu A. (2012). Identification, synthesis and characterization of a novel antimicrobial peptide HKPLP derived from Hippocampus kuda Bleeker. J. Antibiot. (Tokyo).

[73] Georgalaki M.D., Van den Berghe E., Kritikos D., Devreese B., Van Beeumen J., Kalantzopoulos G., De Vuyst L., Tsakalidou E. (2002). Macedocin, a food-grade lantibiotic produced by Streptococcus macedonicus ACA-DC 198. Appl. Environ. Microbiol..

[74] J. Greaves, J. Roboz, Mass spectrometry for the novice, 2013. 10.1201/b15436.

[75] Cavaco M., Valle J., Flores I., Andreu D., R B Castanho M.A. (2021). Estimating peptide half-life in serum from tunable, sequence-related physicochemical properties. Clin. Transl. Sci..

[76] Ebbensgaard A., Mordhorst H., Overgaard M.T., Aarestrup F.M., Hansen E.B. (2018). Dissection of the antimicrobial and hemolytic activity of Cap18: generation of Cap18 derivatives with enhanced specificity. PLoS. One.

[77] Torcato I.M., Huang Y.-H., Franquelim H.G., Gaspar D.D., Craik D.J., Castanho M.A.R.B., Henriques S.T. (2013). The antimicrobial activity of Sub3 is dependent on membrane binding and cell-penetrating ability. Chembiochem..

[78] Zhao Y., Zhang M., Qiu S., Wang J., Peng J., Zhao P., Zhu R., Wang H., Li Y., Wang K., Yan W., Wang R. (2016). Antimicrobial activity and stability of the d-amino acid substituted derivatives of antimicrobial peptide polybia-MPI. AMB Express..

[79] Bahar A.A., Ren D. (2013). Antimicrobial peptides. Pharmaceuticals. (Basel).

[80] Mishra B., Narayana J.Lakshmaiah, Lushnikova T., Wang X., Wang G. (2019). Low cationicity is important for systemic in vivo efficacy of database-derived peptides against drug-resistant Gram-positive pathogens. Proc. Natl. Acad. Sci. U. S. A..

[81] Travkova O.G., Moehwald H., Brezesinski G. (2017). The interaction of antimicrobial peptides with membranes. Adv. Colloid. Interface Sci..

[82] Lee S.-H., Kim S.-J., Lee Y.-S., Song M.-D., Kim I.-H., Won H.-S. (2011). De novo generation of short antimicrobial peptides with simple amino acid composition. Regul. Pept..

[83] Dom G., Shaw-Jackson C., Matis C., Bouffioux O., Picard J.J., Prochiantz A., Mingeot-Leclercq M.-P., Brasseur R., Rezsohazy R. (2003). Cellular uptake of Antennapedia Penetratin peptides is a two-step process in which phase transfer precedes a tryptophan-dependent translocation. Nucleic. Acids. Res..

[84] Andreu D., Merrifield R.B., Steiner H., Boman H.G. (1985). N-Terminal analogs of cecropin A: synthesis, antibacterial activity, and conformational properties. Biochemistry.

[85] Sandgren S., Wittrup A., Cheng F., Jönsson M., Eklund E., Busch S., Belting M. (2004). The human antimicrobial peptide LL-37 transfers extracellular DNA plasmid to the nuclear compartment of mammalian cells via lipid rafts and proteoglycan-dependent endocytosis. J. Biol. Chem..

[86] Langel Ü. (2021). Cell-penetrating peptides and transportan. Pharmaceutics..

[87] Cavaco M., Fraga P., Valle J., Silva R.D.M., Gano L., Correia J.D.G., Andreu D., Castanho M.A.R.B., Neves V. (2024). Molecular determinants for brain targeting by peptides: a meta-analysis approach with experimental validation. Fluids. Barriers. CNS..

[88] Tong S.Y.C., Davis J.S., Eichenberger E., Holland T.L., Fowler V.G. (2015). Staphylococcus aureus infections: epidemiology, pathophysiology, clinical manifestations, and management. Clin. Microbiol. Rev..

[89] Zhang K., Zhang H., Gao C., Chen R., Li C. (2020). Antimicrobial Mechanism of pBD2 against Staphylococcus aureus. Molecules..

[90] Mohamed M.F., Hamed M.I., Panitch A., Seleem M.N. (2014). Targeting methicillin-resistant Staphylococcus aureus with short salt-resistant synthetic peptides. Antimicrob. Agents Chemother.

[91] Spivak E.S., Hanson K.E. (2018). Candida auris: an emerging fungal pathogen. J. Clin. Microbiol..

[92] Pushpanathan M., Rajendhran J., Jayashree S., Sundarakrishnan B., Jayachandran S., Gunasekaran P. (2012). Identification of a novel antifungal peptide with chitin-binding property from marine metagenome. Protein Pept. Lett..

[93] Struyfs C., Cammue B.P.A., Thevissen K. (2021). Membrane-interacting antifungal peptides. Front. Cell Dev. Biol..

[94] Lockhart S.R. (2019). Candida auris and multidrug resistance: defining the new normal. Fungal. Genet. Biol..

[95] Torres R., Barreto-Santamaría A., Arévalo-Pinzón G., Firacative C., Gómez B.L., Escandón P., Patarroyo M.A., Muñoz J.E. (2023). In vitro antifungal activity of three synthetic peptides against Candida auris and other Candida species of medical importance. Antibiotics.

[96] Pathirana R.U., Friedman J., Norris H.L., Salvatori O., McCall A.D., Kay J., Edgerton M. (2018). Fluconazole-resistant Candida auris is susceptible to salivary histatin 5 killing and to intrinsic host defenses. Antimicrob. Agents Chemother.

[97] Puri S., Edgerton M. (2014). How does it kill?: understanding the Candidacidal mechanism of salivary Histatin 5. Eukaryot. Cell.

[98] Xu T., Levitz S.M., Diamond R.D., Oppenheim F.G. (1991). Anticandidal activity of major human salivary histatins. Infect. Immun..

[99] Rather I.A., Sabir J.S.M., Asseri A.H., Ali S. (2022). Antifungal activity of human Cathelicidin LL-37, a membrane disrupting peptide, by triggering oxidative stress and cell cycle arrest in Candida auris. J. Fungi. (Basel).

[100] Büyükkiraz M.Erdem, Kesmen Z. (2022). Antimicrobial peptides (AMPs): a promising class of antimicrobial compounds. J. Appl. Microbiol..

[101] Hoskin D.W., Ramamoorthy A. (2008). Studies on anticancer activities of antimicrobial peptides. Biochim. Biophys. Acta.

[102] Oddo A., Hansen P.R., Hansen P.R. (2017). Antimicrobial Peptides: Methods and Protocols.

[103] Fathi F., Ghobeh M., Shirazi F.H., Tabarzad M. (2023). Design and evaluation of a novel anti-microbial peptide from Cathelicidin-2: selectively active against Acinetobacter baumannii. Iran. J. Pharm. Res..

[104] van der Weerden N.L., Bleackley M.R., Anderson M.A. (2013). Properties and mechanisms of action of naturally occurring antifungal peptides. Cell Mol. Life Sci..

[105] Gubler D.J. (2001). Human arbovirus infections worldwide. Ann. N. Y. Acad. Sci..

[106] Gould E., Pettersson J., Higgs S., Charrel R., de Lamballerie X. (2017). Emerging arboviruses: why today?. One Health.

[107] Sukhralia S., Verma M., Gopirajan S., Dhanaraj P.S., Lal R., Mehla N., Kant C.R. (2019). From dengue to Zika: the wide spread of mosquito-borne arboviruses. Eur. J. Clin. Microbiol. Infect. Dis..

[108] da Mata É.C.G., Mourão C.B.F., Rangel M., Schwartz E.F. (2017). Antiviral activity of animal venom peptides and related compounds. J. Venom. Anim. Toxins. Incl. Trop. Dis..

[109] Lalani S., Gew L.T., Poh C.L. (2021). Antiviral peptides against Enterovirus A71 causing hand, foot and mouth disease. Peptides..

[110] Berkhout B., Eggink D., Sanders R.W. (2012). Is there a future for antiviral fusion inhibitors?. Curr. Opin. Virol..

[111] Baldwin C.E., Sanders R.W., Deng Y., Jurriaans S., Lange J.M., Lu M., Berkhout B. (2004). Emergence of a drug-dependent human immunodeficiency virus type 1 variant during therapy with the T20 fusion inhibitor. J. Virol..

[112] Rimsky L.T., Shugars D.C., Matthews T.J. (1998). Determinants of human immunodeficiency virus type 1 resistance to gp41-derived inhibitory peptides. J. Virol..

[113] Greenberg M.L., Cammack N. (2004). Resistance to enfuvirtide, the first HIV fusion inhibitor. J. Antimicrob. Chemother.

[114] Rothan H.A., Bahrani H., Shankar E.M., Rahman N.A., Yusof R. (2014). Inhibitory effects of a peptide-fusion protein (Latarcin-PAP1-Thanatin) against chikungunya virus. Antiviral Res..

[115] Vilas Boas L.C.P., Campos M.L., Berlanda R.L.A., de Carvalho Neves N., Franco O.L. (2019). Antiviral peptides as promising therapeutic drugs. Cell Mol. Life Sci..

[116] Skalickova S., Heger Z., Krejcova L., Pekarik V., Bastl K., Janda J., Kostolansky F., Vareckova E., Zitka O., Adam V., Kizek R. (2015). Perspective of use of antiviral peptides against influenza virus. Viruses..

[117] Carballar-Lejarazú R., Rodríguez M.H., de la Cruz Hernández-Hernández F., Ramos-Castañeda J., Possani L.D., Zurita-Ortega M., Reynaud-Garza E., Hernández-Rivas R., Loukeris T., Lycett G., Lanz-Mendoza H. (2008). Recombinant scorpine: a multifunctional antimicrobial peptide with activity against different pathogens. Cell Mol. Life Sci..

[118] M. Piatek, G. Sheehan, K. Kavanagh, Galleria mellonella: the versatile host for drug discovery, In Vivo Toxicity Testing and Characterising Host-Pathogen Interactions, Antibiotics (Basel) 10 (2021) 1545. 10.3390/antibiotics10121545. PMC8698334 34943757

[119] Maguire R., Duggan O., Kavanagh K. (2016). Evaluation of Galleria mellonella larvae as an in vivo model for assessing the relative toxicity of food preservative agents. Cell Biol. Toxicol..

[120] Kelly J., Kavanagh K. (2011). Caspofungin primes the immune response of the larvae of Galleria mellonella and induces a non-specific antimicrobial response. J. Med. Microbiol..

[121] Tsai C.J.-Y., Loh J.M.S., Proft T. (2016). Galleria mellonella infection models for the study of bacterial diseases and for antimicrobial drug testing. Virulence.

[122] Kavanagh K., Sheehan G. (2018). The use of galleria mellonella larvae to identify novel antimicrobial agents against fungal species of medical interest. J. Fungi. (Basel).

[123] Fedhila S., Buisson C., Dussurget O., Serror P., Glomski I.J., Liehl P., Lereclus D., Nielsen-LeRoux C. (2010). Comparative analysis of the virulence of invertebrate and mammalian pathogenic bacteria in the oral insect infection model Galleria mellonella. J. Invertebr. Pathol..

[124] Moreira Brito J.C., Carvalho L.R., Neves de Souza A., Carneiro G., Magalhães P.P., Farias L.M., Guimarães N.R., Verly R.M., Resende J.M., Elena de Lima M. (2022). PEGylation of the antimicrobial peptide LyeTx I-b maintains structure-related biological properties and improves selectivity. Front. Mol. Biosci..

[125] Domingues M.M., Inácio R.G., Raimundo J.M., Martins M., Castanho M.A.R.B., Santos N.C. (2012). Biophysical characterization of polymyxin B interaction with LPS aggregates and membrane model systems. Biopolymers.

