# Supplementary material for: Design of Natterins-based peptides improves antimicrobial and antiviral activities
Source: Biotechnol Rep (Amst). 2024 Nov 28;45:e00867. doi: 10.1016/j.btre.2024.e00867 (PMC11697409; doi:10.1016/j.btre.2024.e00867)
Supplement: Supplementary file 1 [file mmc1.docx]

***Supplementary information***

**Design of Natterins-Based Peptides Improves Antimicrobial and Antiviral Activities**

Gabrielle L. de Cena¹, Dayane B. Tada^2^, Danilo B. M. Lucchi^3^, Tiago A.A Santos^4^, Montserrat Heras^5^, Maria Juliano^6^, Carla Torres Braconi^3^; Miguel A.RB. Castanho^4^, Mônica Lopes-Ferreira^7^, Katia Conceição¹*

¹Laboratory of Peptide Biochemistry and ^2^Laboratory of Nanomaterials and Nanotoxicology, Universidade Federal de São Paulo (UNIFESP), São José dos Campos, Brasil; ^3^Department of Microbiology, Immunology and Parasitology and; ^4^Department of Biophysics, Escola Paulista de Medicina, (UNIFESP), São Paulo, Brazil; ^5^Instituto de Medicina Molecular, Faculdade de Medicina de Lisboa, Av. Professor Egas Moniz, 1649-028 Lisboa, Portugal; ^6^Departament de Química, Universitat de Girona, Campus Montilivi, 17071 Girona, Spain; ^7^Immunoregulation Unit, Laboratory of Applied Toxinology (CeTICs/FAPESP), Butantan Institute, São Paulo 05503900, Brazil.

* Correspondence: katia.conceicao@unifesp.br; Tel.: +55 12 3924.9500


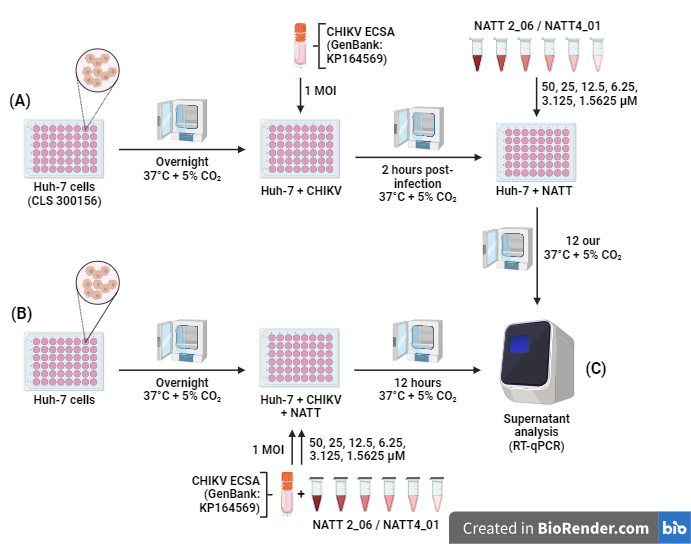


**SFigure 1.** Workflow of the methodology used in antiviral assays. (A) "post-treatment", (B) "co-treatment", (C) viral RNA extraction and molecular characterization. Created in BioRender.com.

**Materials and Methods**

**M1. Synthesis of Natterins based peptides**

M1.2. General remarks

All commercially available chemicals were used as purchased without further purification. Reverse-phase column chromatography purification of NATT peptides was performed on a CombiFlash Rf200 automated flash chromatography system using a RediSep Rf Gold reversed-phase column packed with high-performance C18 derivatized silica (Teledyne ISCO, Lincoln, NE, USA). NATT peptides were analyzed under standard analytical HPLC conditions with a 1260 Infinity II liquid chromatography instrument (Agilent, Santa Clara, CA, USA), using a Kromasil 100 C_18_ (4.6 mm × 40 mm, 3 µm) column with a 2-100% B linear gradient over 17 min at a flow rate of 1 mL min^-1^. Solvent A was 0.1% aqueous TFA, and solvent B was 0.1% TFA in CH_3_CN. Detection was carried out at 220 nm. ESI-HRMS analyses of NATT peptides were performed with a compact mass spectrometer from Bruker Daltonics (Serveis Tècnics de Recerca, University of Girona, STR-UdG). The instrument is equipped with an electrospray ionization (ESI) source with a hybrid quadrupole-time-of-flight (QTOF) analyser. The instrument was operated in the positive ESI(+) ion mode, in a range of 50 to 3000 m/z. Samples were dissolved and they were introduced (1-5 μL) to the spectrometer through an Agilent Technologies 1200 Series HPLC automatic injector at a flow rate of 0.1 mL/min. Nitrogen was employed as drying and nebulising gas. Results were analysed with Bruker Compass DataAnalysis 6.1 software. NMR experiments were performed in STR-UdG with an Ultrashield ASCEND Nanobay 400 instrument (9.4 T) from Bruker (^1^H-NMR, 400 MHz). Structural assignments were made with additional information from COSY experiments. NMR spectra were processed and analyzed using TopSpin 3.6.2. Chemical shifts were reported as δ (parts per million) directly calibrated with the solvent sign. All ^1^H-NMR spectra were recorded in CD_3_OD and referenced to residual CH_3_OH at 3.31 ppm. Coupling constants (*J*) are given in Herst (Hz). The following abbreviations were used for spin multiplicity: d = doblet, t = triplet, quin = quintet, m = multiplet, dd = doublet of doublets.

**M1.3. Solid Phase Synthesis of NATT peptides. General Procedure**

The synthesis of NATT peptides was performed manually on solid phase following a 9‑fluorenylmethoxycarbonyl (Fmoc)/*tert*-butyl (*^t^*Bu) protocol, in polypropylene syringes fitted with a polyethylene porous disk. Solvents and soluble reagents were removed in vacuum. Fmoc-Rink-MBHA resin (0.71 mmol/g) was used as solid support since it provides C-terminal peptides amides. Fmoc group removal was achieved with piperidine-DMF (3:7, 2 + 10 min). Coupling of commercial Fmoc-amino acids (4 or 3 equiv) were performed using DIC (4 or 3 equiv) and Oxima (4 or 3 equiv) in DMF under stirring at room temperature for 4 or 8 h, and monitoring by Kaiser test or Chloramil test for proline residues. For each coupling and deprotection step, the resin was washed with DMF (6 x 1 min) and CH_2_Cl_2_ (3 x 1 min), and aired-dried. After coupling of ninth amino acid residue, NMP was used instead DMF. Peptide elongation was performed by repeated cycles of Fmoc removal, coupling and washings. Once the synthesis was completed, peptidyl resins were subjected to the N-terminal Fmoc removal. Then, the peptides were cleaved by treatment with TFA-H_2_O-TIS (95:2.5:2.5) for 2 h. Following TFA evaporation and diethyl ether extraction, the crude peptides were purified by reverse-phase column chromatography on a CombiFlash Rf200 automated flash chromatography system, lyophilized, analyzed by HPLC, and characterized by high resolution mass spectrometry (HRMS) and proton nuclear magnetic resonance (1H-NMR).

**M1.4. Kaiser test procedure**

The Kaiser or nynhidrin test is a colorimetric and qualitative test that allows the detection of free primary amines. It is used to monitor the coupling of an amino acid to a peptidyl resin. To perform the test, a portion of resin is placed in an Eppendorf and 2-3 drops of the solutions A (2.5 g ninhydrin / 50 mL ethanol), B (0.2 g phenol/ 50 mL ethanol) and C (NaCN 1 mM in pyridine), are added consecutively. The suspension is heated for 3 min at 100 °C and the color of the resin is checked. If the coupling is not complete and free amino groups remain, the solution acquires a blue color (positive test); otherwise, the color is yellow (negative test).

**M1.5. Chloranil test procedure**

Chloranil test is a colorimetric test similar to Kaiser test used for amino acids bearing a secondary amine, such as proline, to monitor the coupling to a peptidyl resin. To perform the test, a portion of resin is placed in an Eppendorf and 4 drops of the solutions A (0.1 mL acetaldehyde / 4.9 mL DMF) and B (0.1 g chloranil / 4.9 g DMF) are added consecutively. The suspension is incubated for 5 min at room temperature and the color of the resin is checked. If the coupling is not complete and free amino groups remain, the solution acquires a blue color (positive test); otherwise, the color is yellow (negative test).

**M1.6. Synthesis of NATT2_06**

Following the general procedure described above, NATT2_06 (TTLRPKLKSK-NH_2_) was obtained in >99% purity HPLC (λ=220 nm): *t*_R_ 3.28. ESI-HRMS (*m/z*) calculated for C_52_H_99_N_17_O_13_, M+nH; [M+2H]^2+^ 585.8877 found 585.8871, [M+3H]^3+^ 390.9276 found 390.9279, [M+4H]^4+^ 293.4475 found 293.4478, [M+5H]^5+^ 234.9594 found 234.9598. ^1^H-NMR (400 MHz, CD_3_OD) δ 0.90 (d, *J*=6.2 Hz, 3H, C*H_3δ_*-Leu), 0.92 (d, *J*=5.8 Hz, 3H, C*H_3δ_*‑Leu), 0.95 (d, *J*=6.5 Hz, 3H, C*H_3δ_*-Leu), 0.97 (d, *J*=6.3 Hz, 3H, C*H_3δ_*-Leu), 1.24 (d, *J*=6.4 Hz, 3H, C*H_3γ_*-Thr^2^), 1.32 (d, *J*=6.4 Hz, 3H, C*H_3γ_*-Thr^1^), 1.42-2.12 (m, 31H, 1 proton C*H_2β_*-Lys^10^ + 2xC*H_2β_*-Lys + 3xC*H_2γ_*-Lys + 3xC*H_2δ_*-Lys + C*H_2β_*-Arg + C*H_2γ_*-Arg + C*H_2β_*-Pro + C*H_2γ_*-Pro + 2xC*H_2β_*-Leu + 2xC*H_γ_*-Leu), 2.25 (m, 1H, 1 proton C*H_2β_*-Lys^10^), 2.89-3.00 (m, 6H, 3xC*H_2ε_*-Lys), 3.12-3.28 (m, 2H, C*H_2δ_*-Arg), 3.66 (m, 1H, 1 protó C*H_2δ_*-Pro), 3.74 (m, 1H, *H_δ_*-Pro)^[[1]](#footnote-1)^, 3.78 (dd, *J*=11.0 Hz, *J’*=5.7 Hz, 1H, 1 proton C*H_2β_*‑Ser), 3.87-3.94 (m, 2H, 1 proton C*H_2β_*-Ser + *CH_a_*-Thr^1^), 4.15 (quin, *J*=6.2 Hz, 1H, C*H_β_*-Thr^1^), 4.21 (m, 1H, C*H_β_*-Thr^2^), 4.27-4.44 (m, 8H, 3xC*H_α_*-Lys + 2xC*H_α_*-Leu + C*H_α_*-Thr^2^ + C*H_α_*-Arg + C*H_α_*-Ser), 4.60 (m, 1H, C*H_α_*-Pro).

**M1.7. Synthesis of NATT4_01**

Following the general procedure described above, NATT4_01 (LYVAKNKYGLGKL-NH_2_) was obtained in >99% purity HPLC (λ=220 nm): *t*_R_ 4.31. ESI-HRMS (*m/z*) calculated for C_70_H_116_N_18_O_16_, M+nH; [M+2H]^2+^ 733.4481 found 733.4472, [M+3H]^3+^ 489.3012 found 489.3004, [M+4H]^4+^ 367.2277 found 367.2278. ^1^H-NMR (400 MHz, CD_3_OD) δ 0.89-0.99 (m, 24H, 6xC*H_3β_*-Leu + 2xC*H_3γ_*-Val), 1.10-1.27 (m, 2H, 1xC*H_2γ_*_-_Lys), 1.39 (d, *J*=7.4 Hz, 3H, C*H_3β_*-Ala), 1.42-1.50 (m. 4H, 2xC*H_2γ_*-Lys), 1.51-1.92 (m, 21H, 3xC*H_2β_*-Lys + 3xC*H_2δ_*-Lys + 3xC*H_2β_*-Leu + 3xC*H_γ_*-Leu), 2.03 (octet, *J*=6.9 Hz, C*H_β_*-Val), 2.79-2.97 (m, 10H, 1xC*H_2β_*-Tyr^2^ + 1xC*H_2β_*-Tyr^8^ + C*H_2β_*-Asp + 3xC*H_2ε_*-Lys), 3.04 (dd, *J*=14.0 Hz, *J’*=5.0 Hz, 1xC*H_2β_*‑Tyr), 3.16 (dd, *J*=14.0 Hz, 1H, *J’*=6.6 Hz, 1xC*H_2β_*-Tyr), 3.79 (d, *J*=16.5 Hz, 1H, 1xC*H_2α_*-Gly^A^), 3.84-3.90 (m, 3H, C*H_2α_*-Gly^B^ + C*H_α_*-Leu^1^), 3.98 (d, *J*=16.5 Hz, 1H, 1xC*H_2α_*-Gly^A^), 4.07 (t, *J*=6.7 Hz, 1H, C*H_α_*-Leu^10^). 4.16 (d, 1H, C*H_α_*-Val), 4.27-4.41 (m, 5H, 3xC*H_α_*-Lys + C*H_α_*-Leu^13^ + C*H_α_*-Ala), 4.48 (dd, *J*=10.4 Hz, *J’*=5.1 Hz, 1H, C*H_α_*-Tyr), 4.67 (m, 2H, C*H_α_*-Tyr + C*H_α_*-Asn), 6.69 (d, *J*=8.4 Hz, 2H, 2xC*H_orto_*‑(OH)Tyr)^[[2]](#footnote-2)^, 6.71 (d, *J*=8.4 Hz, 2H, 2xC*H_orto_*‑(OH)Tyr), 7.07 (d, *J*= 8.4 Hz, 2H, 2xC*H_meta_*‑(OH)Tyr)^[[3]](#footnote-3)^, 7.08 (d, *J*=8.4 Hz, 2H, 2xC*H_meta_*‑(OH)Tyr).

**
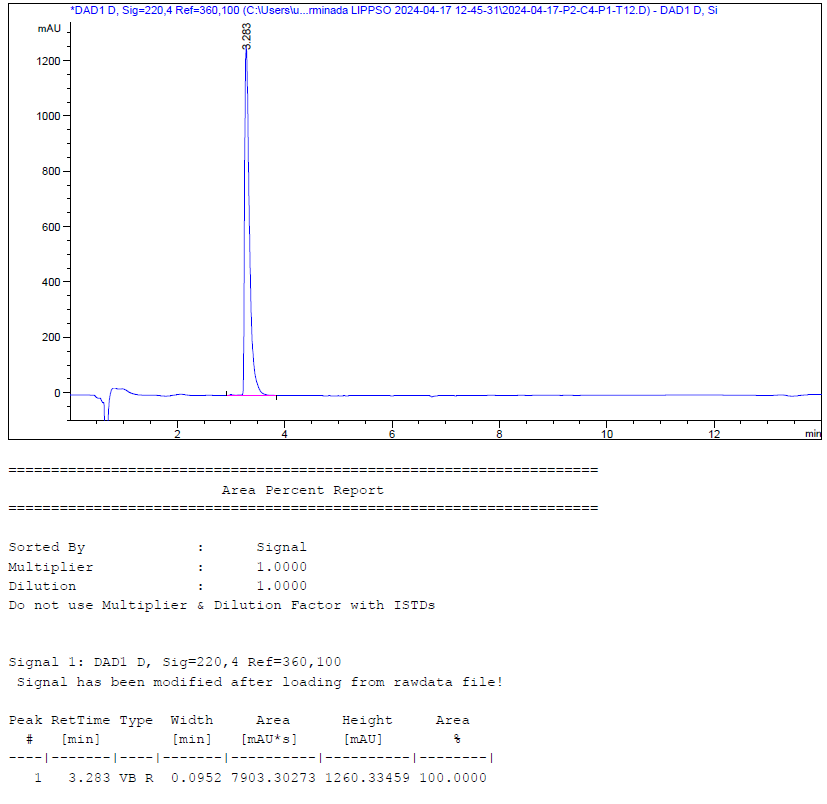
**

**SFigure 2.** Representative Chromatogram of synthesis purification process of NATT2_06 peptide

**SFigure 3.** Representative ESI-HRMS purification process of NATT2_06 peptide.

**A)**


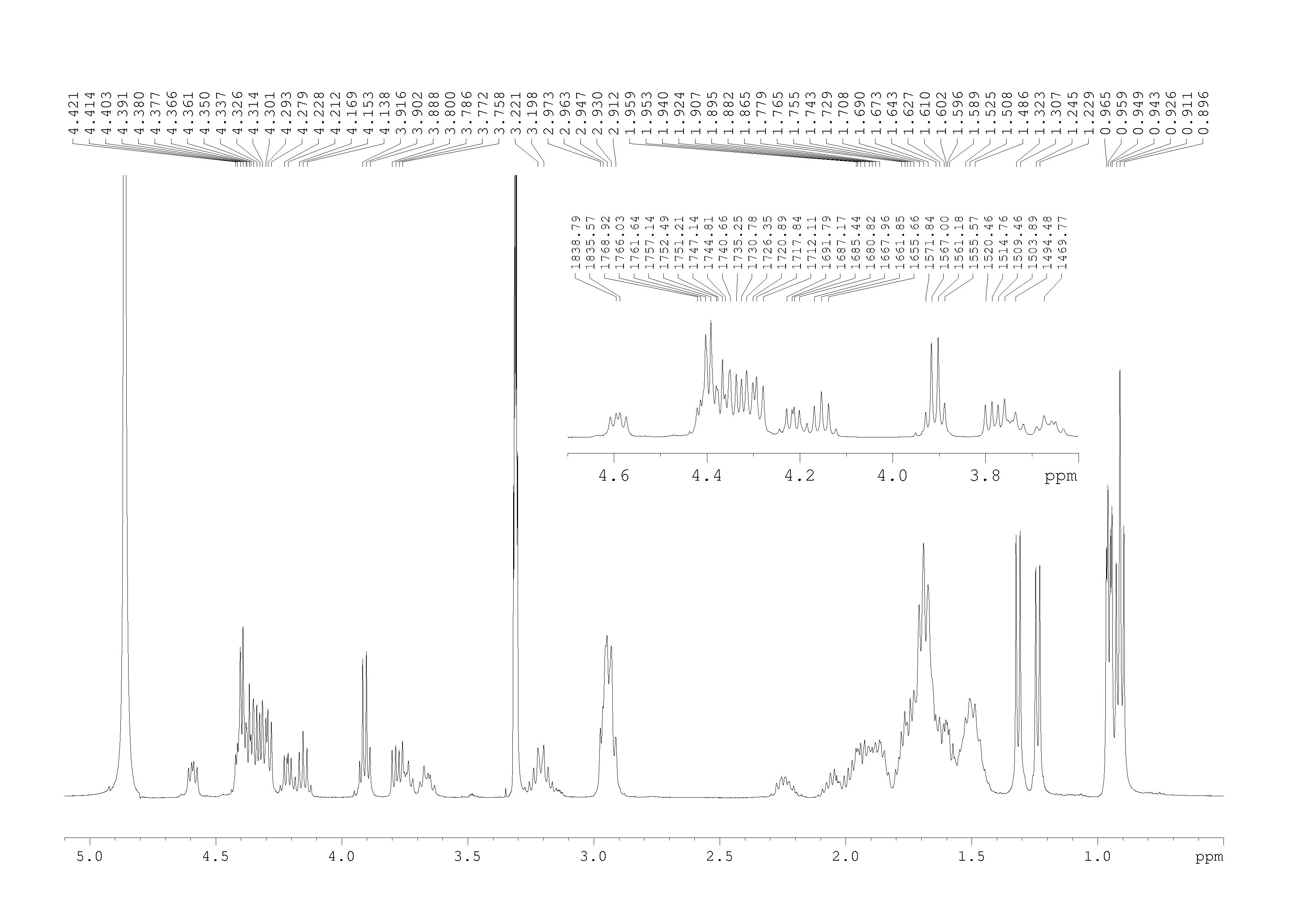

**B)**

**
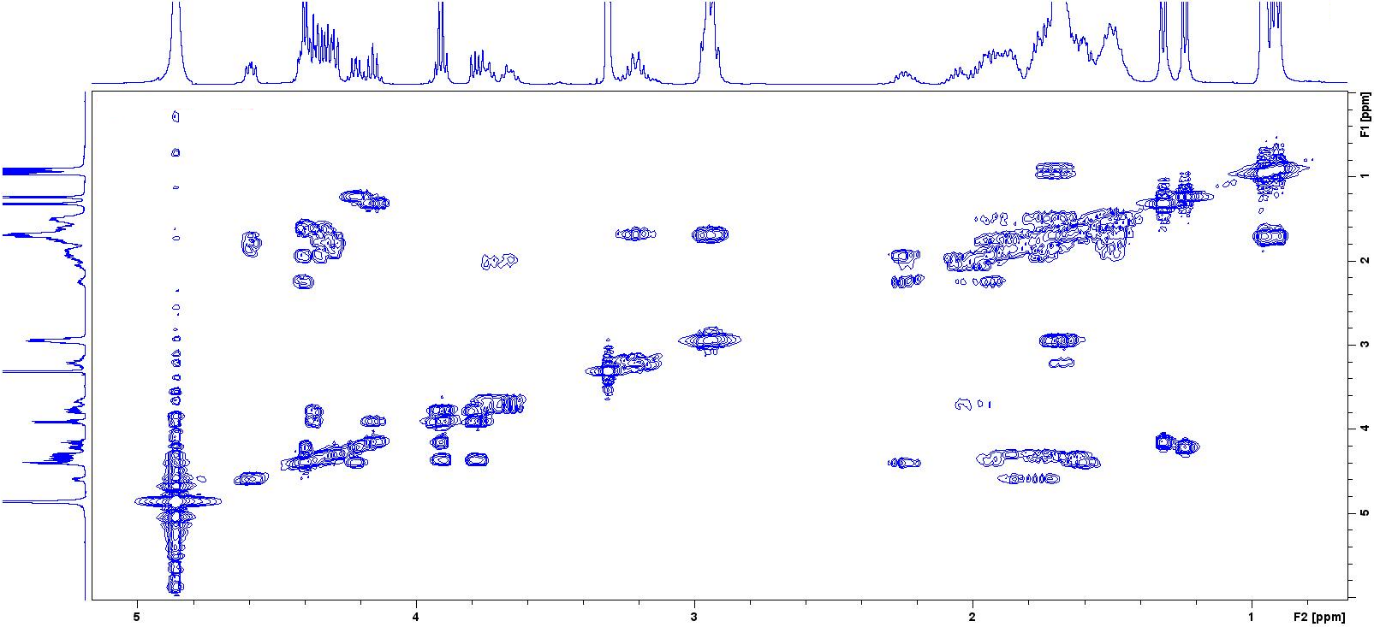
**

**SFigure 4.** Representative (A) ^1^H-NMR and (B) COSY NMR purification process of NATT2_06 peptide.

**SFigure 5.** Representative Chromatogram of synthesis purification process of NATT4_01 peptide

**SFigure 6.** Representative ESI-HRMS purification process of NATT4_01 peptide.


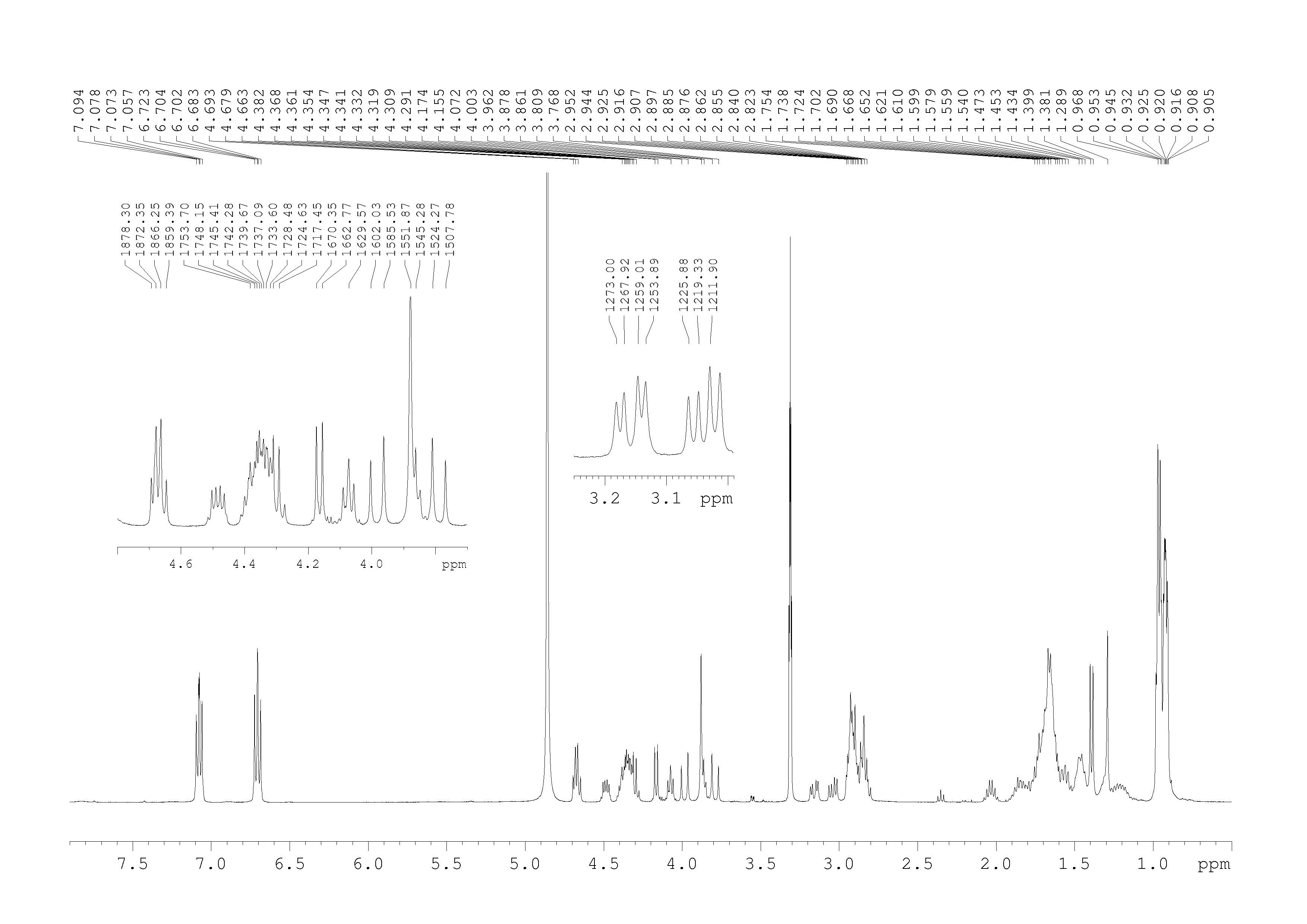

**
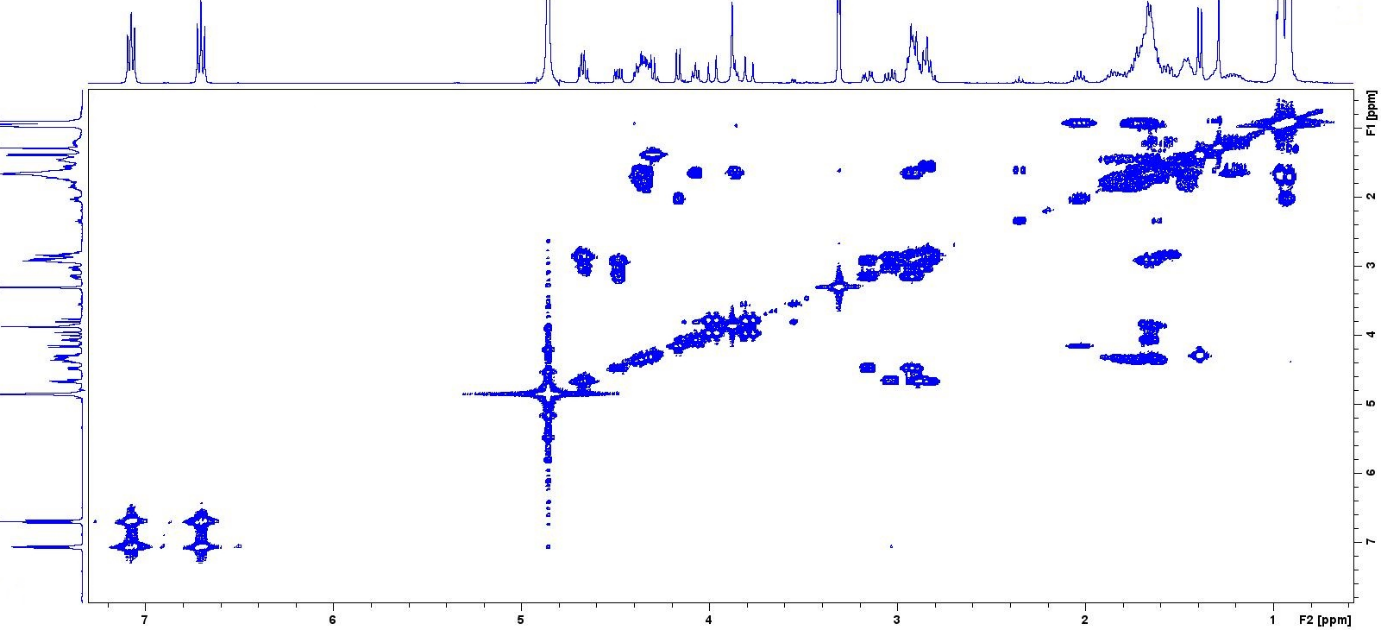
**

**SFigure 7.** Representative (A) ^1^H-NMR and (B) COSY NMR purification process of NATT4_01 peptide.


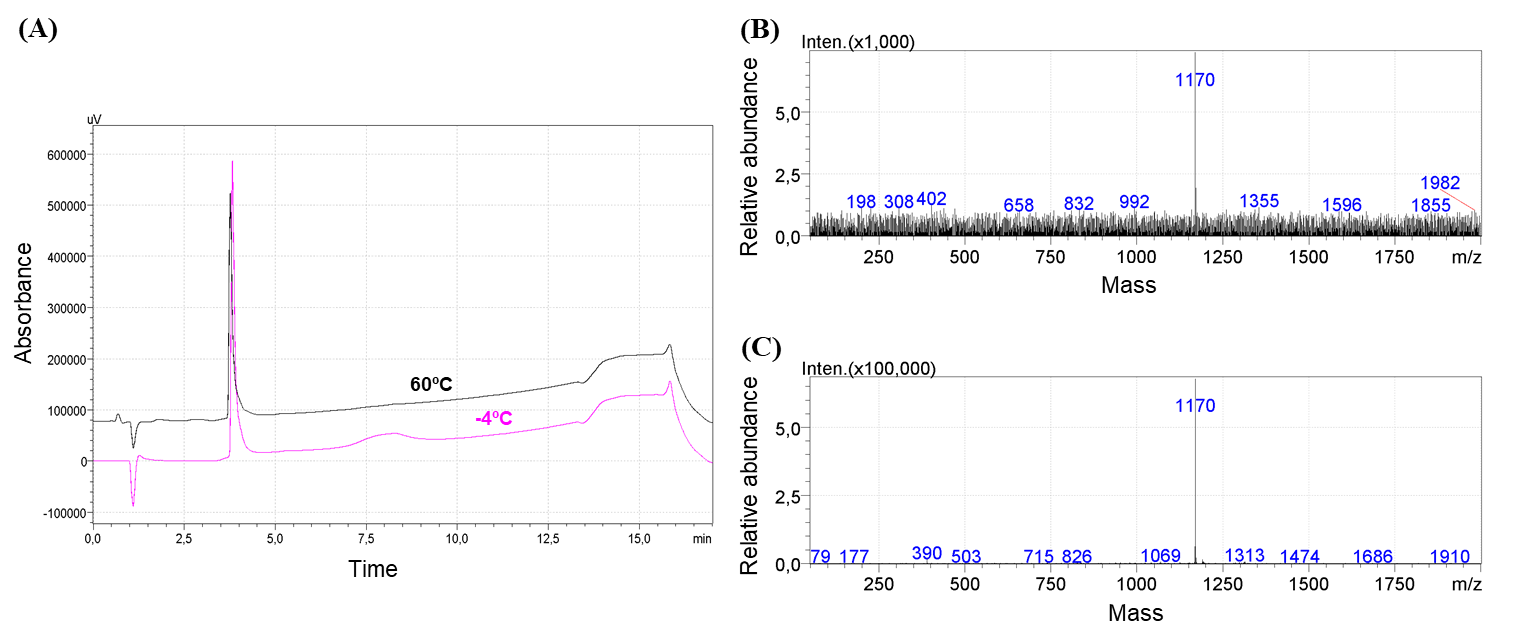


**Figure S8.** Stability analysis of NATT2_06. (A) HPLC chromatogram (pink line - control at -4 °C, black line - analysis at 60 °C), (B) Representative MS spectra of peptide control at -4 °C and (C) MS analysis at 60 °C.


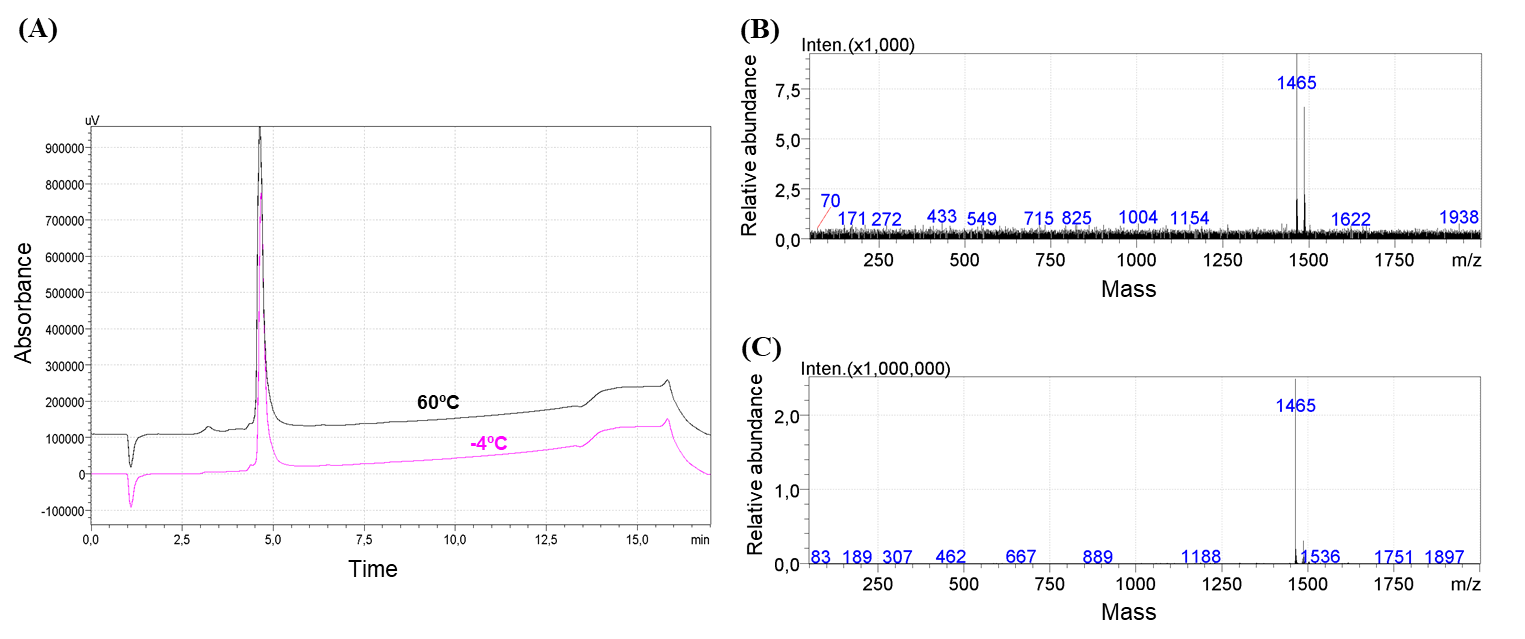


**Figure S9.** Stability analysis of NATT4_01. (A) HPLC chromatogram (pink line - control at -4 °C, black line - analysis at 60 °C); (B) Representative MS spectra of peptide control at -4 °C and (C) MS analysis at 60 °C.


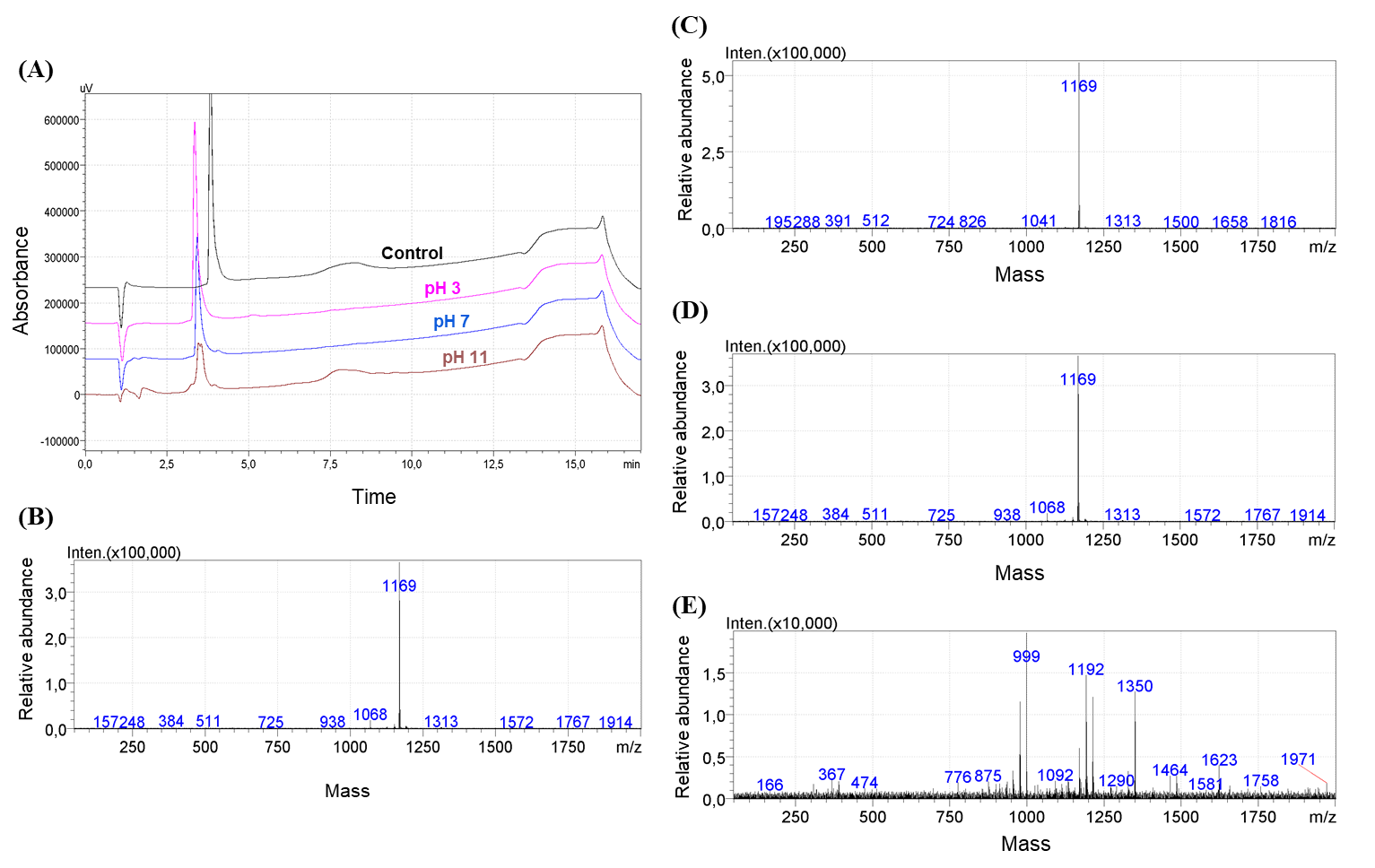


**Figure S10.** Stability analysis of NATT2_06. (A) HPLC chromatogram (black line - control, pink line - pH 3, blue line - pH 7, brown line - pH 11), (B) Representative MS spectra of peptide analysis control; (C) MS analysis at pH 3; (D) MS analysis at pH 7; (E) MS analysis at pH 11.


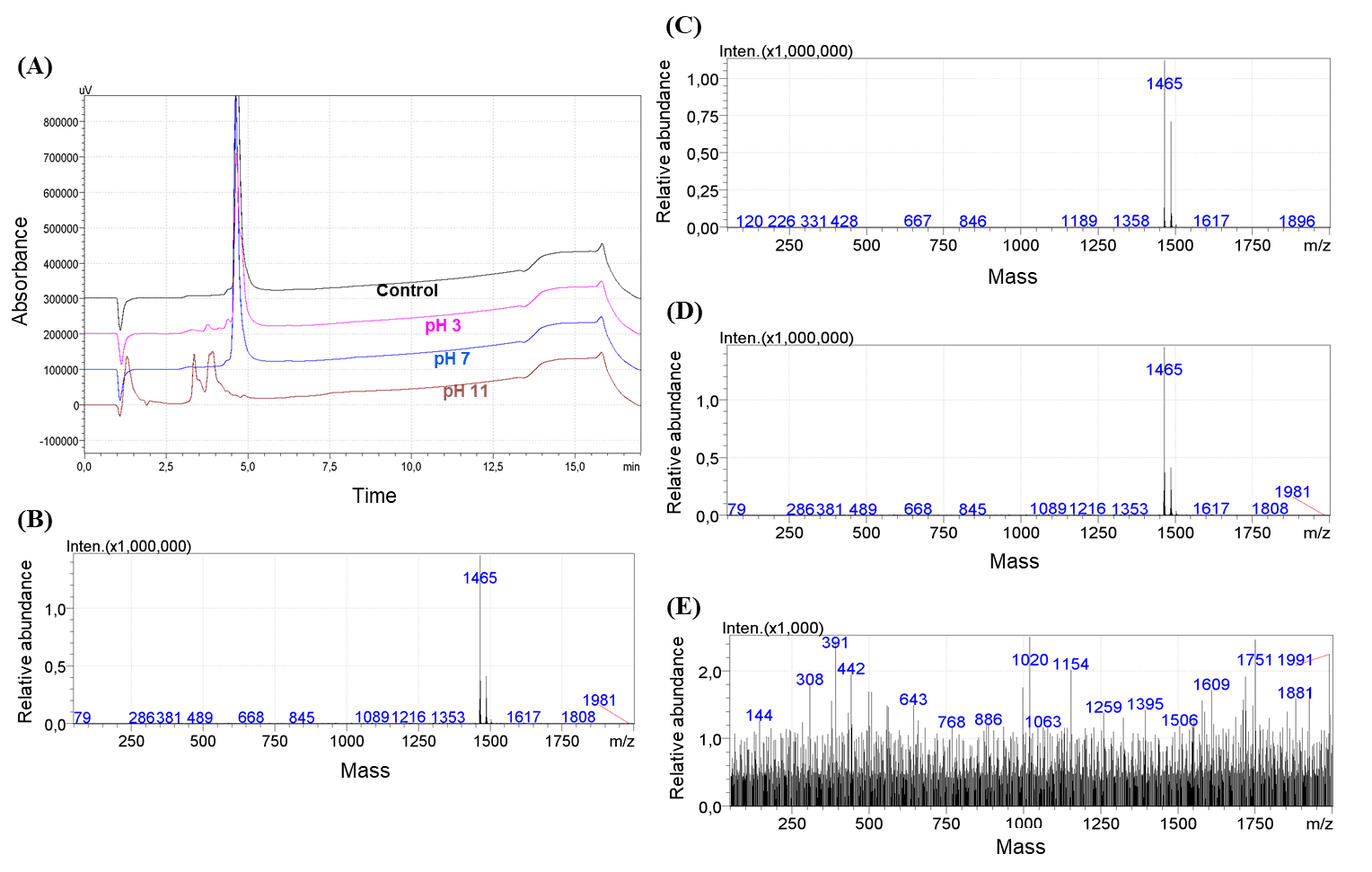


**Figure S11.** Stability analysis of NATT4_01. (A) HPLC chromatogram (black line - control, pink line - pH 3, blue line - pH 7, brown line - pH 11), (B) Representative MS spectra of peptide analysis control; (C) MS analysis at pH 3; (D) MS analysis at pH 7; (E) MS analysis at pH 11.


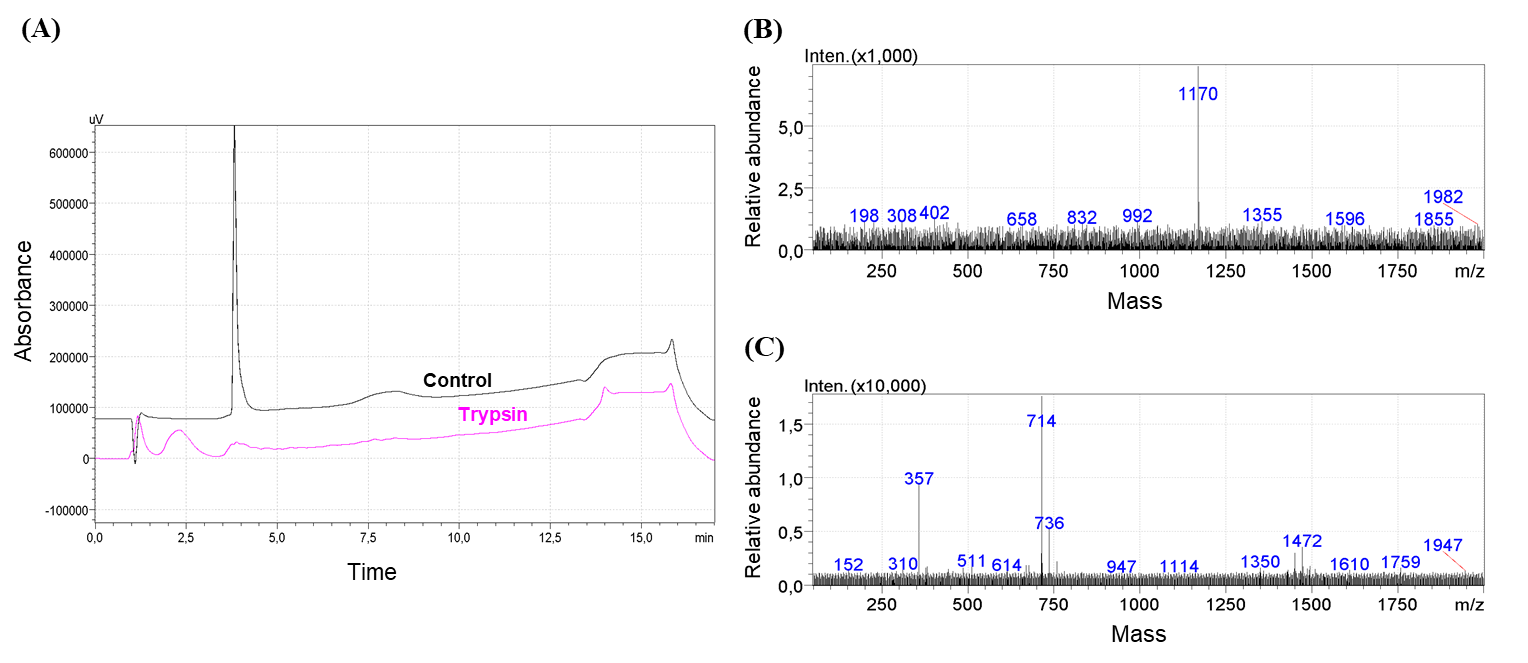


**Figure S12**. Stability analysis of NATT2_06. (A) HPLC chromatogram (black line - control, pink line - trypsin); (B) Representative MS spectra of peptide analysis control; (C) MS analysis of peptide subjected to trypsin.


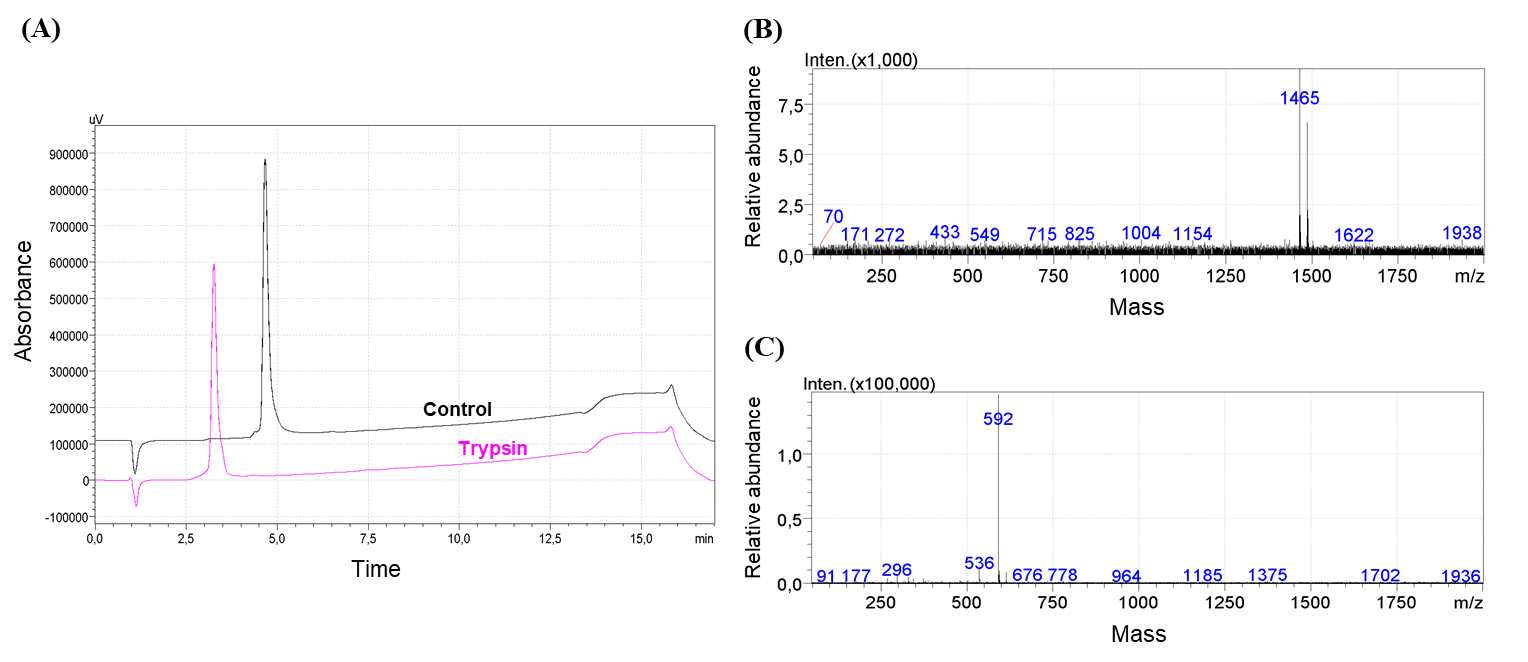


**Figure S13.** Stability analysis of NATT4_01. (A) HPLC chromatogram (black line - control, pink line - trypsin); (B) Representative MS spectra of peptide analysis control; (C) MS analysis of peptide subjected to trypsin.

**STable 1.** Sequences of oligonucleotides and probe used in the assays

| **NAME** | **SEQUENCE** | **AMPLIFIED REGION** |
| --- | --- | --- |
| *Oligonucleotides* |  | |
| **CHIKV_FP** | 5’-YgAYCAYgCMgWCACAg-3’ | E1 Protein |
| **CHIKV_RP** | 5’-AARggYgggTAgTCCATgTT-3’ |  |
| *Probe* |  | |
| **CHIKV_P** | FAM-CCAATGTCYTCMGCCTGGACRCCKTT-MGB-NFQ | |

FP: f*oward primer*, RP: *reverse primer*, P: *probe*.

To assist in the analysis of the results, a standard curve was constructed with the dilution titers (in Log_10_(PFU)) plotted on the x-axis and their respective mean C_T_ values (number of cycles) plotted on the y-axis (Figure 3). Subsequently, a simple linear regression of the points was performed, resulting in a straight line equation y = -2.815x + 27.62 with an R² value of 0.9944. Using the linear equation, the conversion of C_T_ values from the plaque challenge assays was carried out, expressed as the mean Log_10_(PFU/mL) ± S.D (standard deviation of the mean).


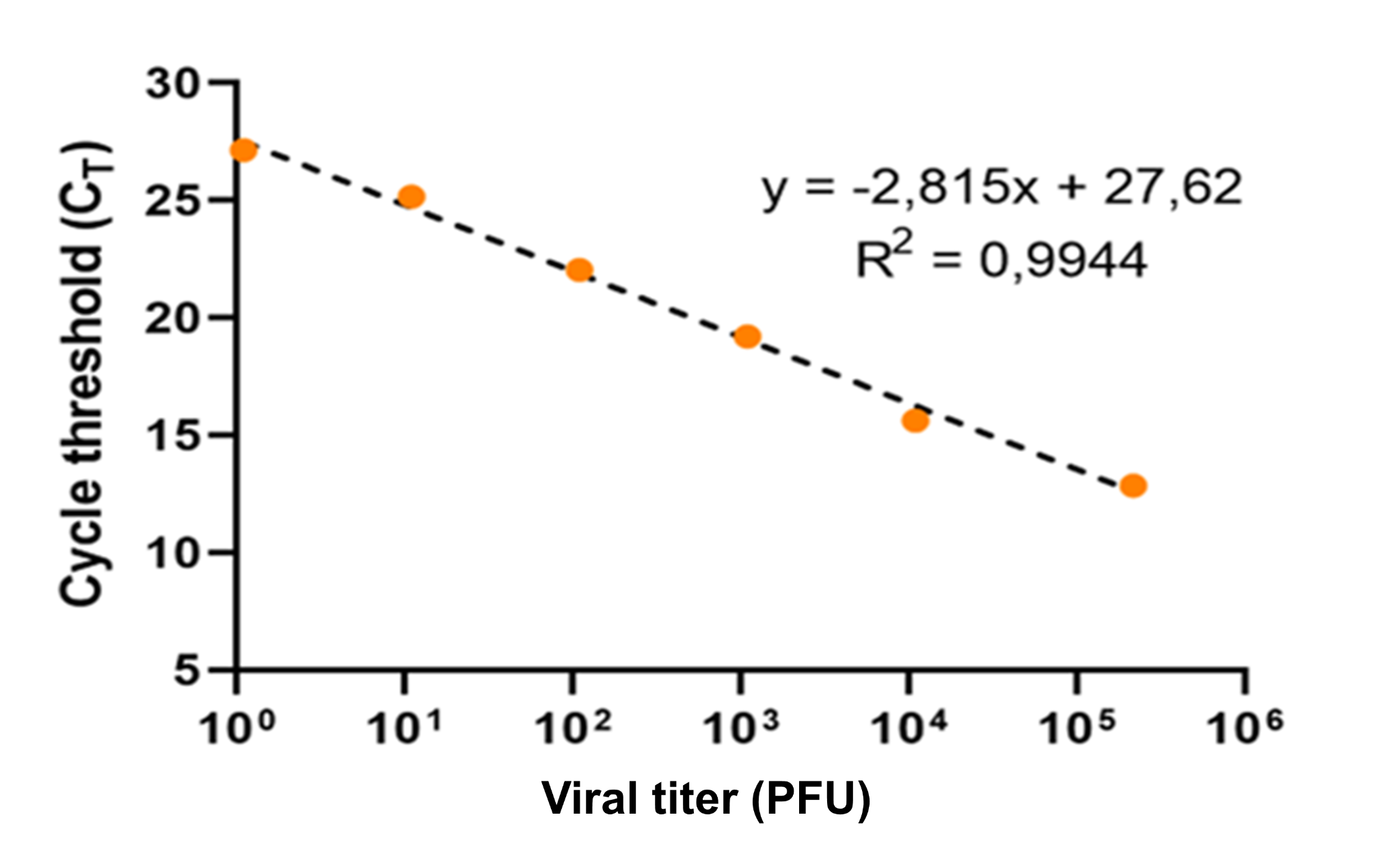


**SFigure 14.** Standard curve for CHIKV quantification. The x-axis represents plaque-forming units (PFU) in Log_10_, and the y-axis represents the mean C_T_ values. The curve was constructed from a simple linear regression of the points.

**STable 2.** Viral loads of Huh-7 cells infected with CHIKV and treated with NATT 2_06 or NATT 4_01 for 12 hours

| **Type of Assay** | **Peptide** | **Concentration (μM)** | **Log_10_(PFU/mL) (Mean ± S.D)** |
| --- | --- | --- | --- |
| “Post-treatment” | NATT 2_06 | 50 | 4.23 ± 0.49 |
|  |  | 25 | 4.65 ± 0.60 |
|  |  | 12.5 | 4.26 ± 0.26 |
|  |  | 6.25 | 4.38 ± 0.52 |
|  |  | 3.125 | 4.36 ± 0.65 |
|  |  | 1.5625 | 5.22 ± 0.73 |
|  |  | Only CHIKV | 5.24 ± 0.83 |
|  |  | Mock | 0 |
|  | NATT 4_01 | 50 | 5.73 ± 0.20 |
|  |  | 25 | 5.19 ± 0.72 |
|  |  | 12.5 | 5.23 ± 0.44 |
|  |  | 6.25 | 5.16 ± 0.69 |
|  |  | 3.125 | 5.50 ± 0.03 |
|  |  | 1.5625 | 5.58 ± 0.12 |
|  |  | Only CHIKV | 5.79± 0.12 |
|  |  | Mock | 0 |
| “Co-treatment” | NATT 2_06 | 50 | 5.18 ± 0.87 |
|  |  | 25 | 5.96 ± 0.32 |
|  |  | 12.5 | 5.76 ± 0.20 |
|  |  | 6.25 | 5.48 ± 0.70 |
|  |  | 3.125 | 6.10 ± 0.47 |
|  |  | 1.5625 | 6.62 ± 0.12 |
|  |  | Only CHIKV | 5.78 ± 0.43 |
|  |  | Mock | 0 |
|  | NATT 4_01 | 50 | 4.31 ± 0.50 |
|  |  | 25 | 4.16 ± 0.12 |
|  |  | 12.5 | 3.94 ± 0.18 |
|  |  | 6.25 | 3.34 ± 0.24 |
|  |  | 3.125 | 4.32 ± 0.74 |
|  |  | 1.5625 | 5.23 ± 0.68 |
|  |  | Only CHIKV | 5.57 ± 0.69 |
|  |  | Mock | 0 |


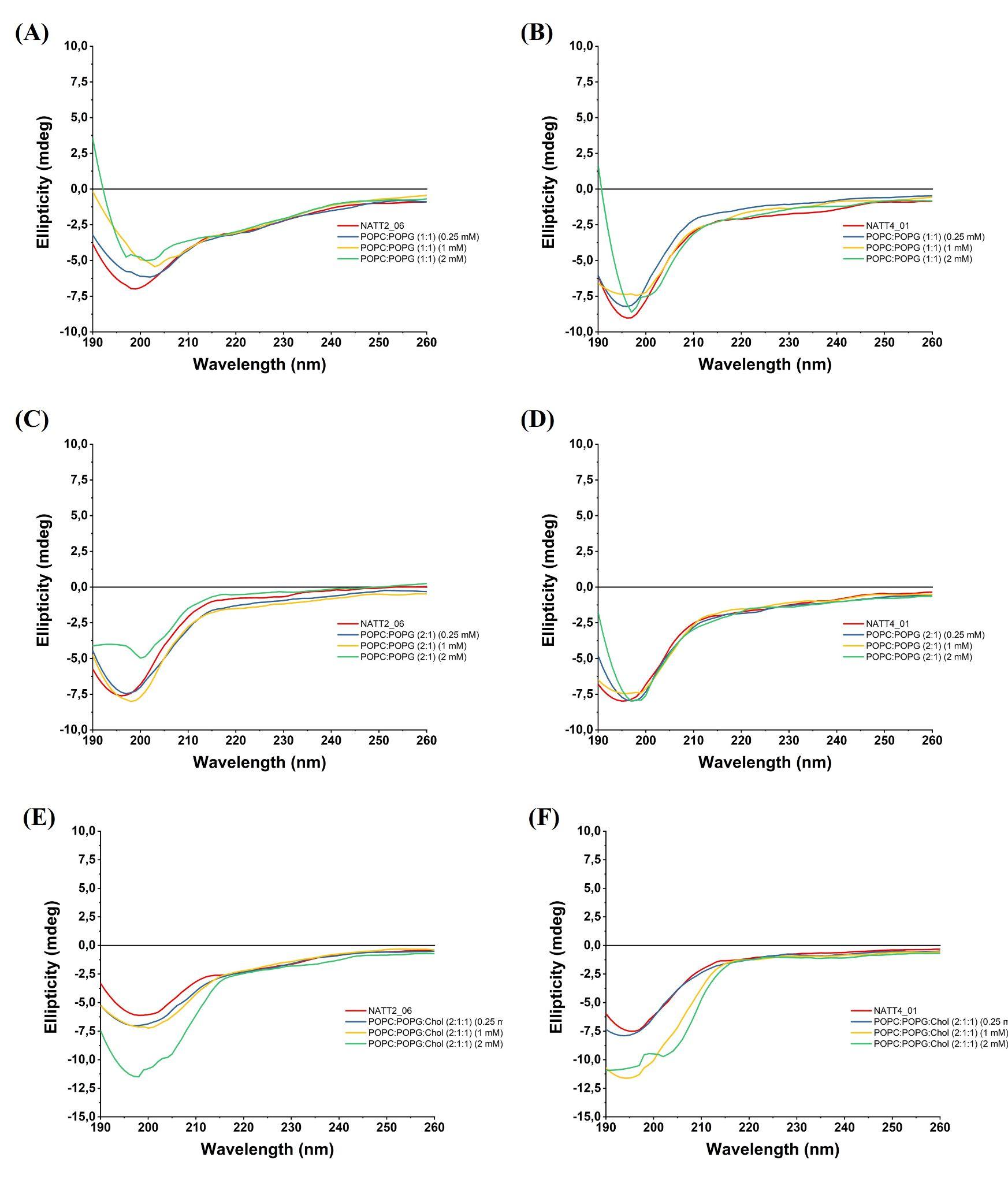


**SFigure 15.** Circular dichroism spectra of peptides (A) NATT2_06 in the absence and presence of different concentrations of POPC : POPG (1:1), (B) NATT4_01 in the absence and presence of different concentrations of POPC : POPG (1:1), (C) NATT2_06 in the absence and presence of different concentrations of POPC : POPG (2:1), (D) NATT4_01 in the absence and presence of different concentrations of POPC : POPG (2:1), (E) NATT2_06 in the absence and presence of different concentrations of POPC : POPG : Cholesterol (2:1:1) and (F) NATT4_01 in the absence and presence of different concentrations of POPC : POPG : Cholesterol (2:1:1).

1. Signal partially overlapped with 3.78 (dd, *J*=11.0 Hz, *J’*=5.7 Hz, 1H, 1 proton C*H_2β_*-Ser). [↑](#footnote-ref-1)
2. Signal partially overlapped with 6.71 (d, *J*=8.4 Hz, 2H, 2xC*H_orto_*‑(OH)Tyr) [↑](#footnote-ref-2)
3. Signal partially overlapped with 7.08 (d, *J*=8.4 Hz, 2H, 2xC*H_meta_*‑(OH)Tyr) [↑](#footnote-ref-3)
